# Organ- and Cell-Specific Induction of the NFE2L2 Antioxidant Programme After Human Graft Reperfusion

**DOI:** 10.3390/biom16101485

**Published:** 2026-10-13

**Authors:** Hyeonah Oh, So Hyun Ahn

**Affiliations:** 1 Department of Chemistry, College of Science, Yonsei University, 50 Yonsei-ro, Seodaemun-gu, Seoul 03722, Republic of Korea; hyeonahoh@yonsei.ac.kr; 2 Ewha Medical Research Institute, School of Medicine, Ewha Womans University, Seoul 03760, Republic of Korea; 3 Ewha Medical Artificial Intelligence Center, School of Medicine, Ewha Womans University, Seoul 03760, Republic of Korea

**Keywords:** reperfusion injury, organ preservation, oxidative stress, transcriptome, organ transplantation

## Abstract

Introduction: Reactive oxygen species at reperfusion are held as a shared injury mechanism across organs, so antioxidant strategies are systemic. Whether the graft’s own response is uniform is untested in human tissue. Methods: Eight public cohorts of paired pre/post-reperfusion human transplant biopsies (kidney, 96 pairs; liver, 40; lung, 54) were analysed with a pre-specified 99-gene antioxidant/ROS panel in ten modules. Within-person differences were compared between organs (paired Wilcoxon, Benjamini–Hochberg) and pooled by random-effects meta-analysis. Two single-cell datasets (lung, 117,549 cells, six recipients; liver, 8802 cells, one donor) resolved cell type. All measures are transcriptional. Results: Antioxidant transcription did not rise as a whole: constitutive scavenging enzyme transcripts fell or were unchanged in every organ. Increase was confined to the NFE2L2-dependent antioxidant programme, graded by organ (lung 98.1%, liver 92.5%, kidney 62.5%; pooled *d_z_* 0.99, *I*^2^ = 89%), with 72.5% of that heterogeneity attributable to organ and no covariate significant after correction. Single-cell data support involvement of epithelium, endothelium, fibroblasts and resident macrophages, but not recruited myeloid cells. Conclusion: At the transcript level the reperfusion response is an inducible programme of graded completeness in resident graft cells. Whether antioxidant intervention must be directed by organs, timing and cell type remains untested.

## 1. Introduction

Organs recovered for transplantation are held in hypothermic storage and then abruptly re-exposed to oxygenated blood. The resulting ischaemia–reperfusion injury contributes to delayed graft function after kidney transplantation, to early allograft dysfunction after liver transplantation and to primary graft dysfunction after lung transplantation, and it shortens graft survival in all three settings [1,2,3].

Reactive oxygen species are central to the accepted account of this injury. Succinate accumulated during ischaemia drives a burst of superoxide from mitochondrial complex I within moments of reoxygenation, and this burst is upstream of much of the sterile inflammation that follows [4,5]. Xanthine oxidoreductase and the NADPH oxidases contribute further oxidant flux [6]. Because these mechanisms are described in comparable terms in the heart, kidney, liver and lung, reactive oxygen species have come to be treated as a mechanism shared across organs, and the therapeutic inference has followed: an antioxidant effective in one organ is expected to be effective in others.

That inference has been tested repeatedly without consistent benefit. N-acetylcysteine, ascorbic acid, hydrogen sulphide donors and free radical scavengers have all been examined in transplantation or in analogous ischaemic settings without establishing a consistent graft benefit [7,8,9,10,11]. Machine perfusion, which changes the conditions of preservation rather than supplementing a single antioxidant, has produced clearer improvements [12,13,14,15]. This contrast raises a question that precedes any choice of agent: what does the graft itself do to its antioxidant machinery when blood returns, and does it do the same thing in every organ?

The endogenous response comprises two arms that are regulated differently. Constitutive scavenging enzymes—superoxide dismutases, catalase, the glutathione peroxidases, the peroxiredoxin and thioredoxin couples—operate continuously and are regulated largely by substrate availability and redox state [16,17,18]. Separately, an inducible programme controlled by NFE2L2 and its repressor KEAP1 raises transcription of haem oxygenase 1, NAD(P)H quinone dehydrogenase 1, the glutamate-cysteine ligase subunits, thioredoxin reductase 1 and the cystine transporter *SLC7A11* [19,20]. Glutathione synthesis and the NADPH supply that regenerates the thiol pools are themselves NFE2L2 targets [21,22,23]. This second arm is referred to throughout as the NFE2L2-dependent antioxidant programme, and is measured here by the NFE2L2 axis module of the gene panel defined below. The two arms need not move together, and a therapy aimed at one is not equivalent to a therapy aimed at the other.

Human data adequate to separate the arms across organs have only recently become available. Several groups have deposited paired biopsies taken from the same graft before and after reperfusion, in kidney [24], liver [25,26,27] and lung [28,29]. Analysed together, and with the within-person design preserved, these cohorts permit a direct comparison of what each organ does at reperfusion.

We therefore asked three questions. Does reperfusion raise antioxidant gene expression as a whole, or only part of it? Does the response differ between kidney, liver and lung? And can any difference be attributed to the organ rather than to the interval at which each organ is sampled or to the platform on which it was measured?

One limitation applies to every result reported here and is stated before any of them. This study measures transcript abundance. Transcript abundance is not a measure of protein abundance, of enzyme activity, or of functional antioxidant capacity, and none of these was assayed in any of the cohorts analysed. Where the term antioxidant defence is used below, it refers to the transcriptional response of the genes that encode that defence, not to the capacity itself.

## 2. Materials and Methods

### 2.1. Data Sources and Cohort Selection

Public repositories were searched for transcriptomic studies of human transplant biopsies with sampling both before and after reperfusion, and with the pairing recoverable from the deposited metadata. Eight cohorts met these conditions and are described in Table 1. Three were designated primary because they provided the largest paired series for their organ: GSE43974 for kidney [24], GSE151648 for liver [25] and GSE268041 for lung [28]. Five further cohorts served for replication and sensitivity analysis: the living-donor arm of GSE43974, three liver cohorts sampled at defined intervals after reperfusion (GSE12720 [26], GSE15480 [27] and GSE23649 [30]) and a second lung cohort (GSE145989 [31]). All were obtained from the Gene Expression Omnibus [32].

Two constraints on cohort selection deserve statement. First, the two lung cohorts share forty-seven of their patient identifiers and are therefore not independent; GSE145989 is reported only as a sensitivity analysis and never as replication, and both are shown separately throughout. Second, GSE23649 and GSE145989 have no linked publication and are cited by accession [30,31].

Pairing in GSE43974 was established from the Supplementary File of the original publication [24], which supplies chip addresses for each pre/post pair; these map one to one onto the sample description field of the series matrix. An index-based alternative was checked against this and does not reproduce it: the index sets for the two time points are not equal, and delayed graft function status, which must agree within a true pair, agreed in only twenty-five of thirty-eight and five of ten candidate pairs in the two deceased-donor strata. That pairing was used throughout.

A note on the liver RNA-sequencing accession: GSE151648 is linked here to [25] via the Gene Expression Omnibus series record itself (PubMed linkage, BioProject and SRA metadata). The accession printed in that article’s text, GSE87487, is a separate and earlier deposit from the same laboratory (20 samples, linked to a different publication) and was not used in this analysis.

### 2.2. Gene Panel

An antioxidant and reactive oxygen species panel was defined before any expression data were examined, and each gene carries the database records that justify its inclusion. Sources were the Reactome pathways for detoxification of reactive oxygen species (R-HSA-3299685), NFE2L2 regulation of antioxidant and detoxification enzymes (R-HSA-9818027), NFE2L2 regulation of pentose phosphate pathway genes (R-HSA-9818028) and nuclear events mediated by NFE2L2 (R-HSA-9759194) [33]; the Gene Ontology terms antioxidant activity (GO:0016209), response to oxidative stress (GO:0006979), cellular oxidant detoxification (GO:0098869) and superoxide anion generation (GO:0042554) [34]; the MSigDB hallmark reactive oxygen species pathway [35]; and HGNC gene family membership [36]. Symbols were normalised to approved HGNC symbols through the alias and previous-symbol tables, and the panel was restricted to protein-coding genes.

The panel comprises ninety-nine genes in ten modules: superoxide dismutases, catalase, glutathione peroxidases, peroxiredoxins, the thioredoxin system, glutathione synthesis and NADPH supply, the NFE2L2 axis, glutathione S-transferases, metallothioneins and genes generating reactive oxygen species. Twenty genes, all glutathione S-transferases or metallothioneins, entered on HGNC family membership alone and are marked as such in the supplied panel file. Including a module for reactive oxygen species generation was deliberate: without it a change in defence cannot be distinguished from a change in the oxidant load that defence answers.

Three terms are used consistently from here on and are not interchangeable. The NFE2L2-dependent antioxidant programme is the biological programme defined in the Introduction. The NFE2L2 axis module is the ten-gene module of this panel that measures it, and is the label used in the tables and figures. The inducible arm is the wider grouping of the NFE2L2 axis module together with the metallothioneins, the two modules that rose at reperfusion; the constitutive arm is the remaining scavenging modules.

### 2.3. Processing

Array cohorts were taken as the deposited normalised matrices and placed on a log_2_ scale where the deposited values were linear. Probes were mapped to approved symbols through the platform annotation, and where several probes matched one gene the probe with the highest mean signal was retained. The liver RNA-sequencing cohort was taken as the deposited count matrix, mapped from Ensembl identifiers, filtered to genes with more than one count per million in at least twenty libraries, and expressed as log_2_ counts per million. Where several rows of a deposited matrix carried the same gene symbol, the row with the highest mean signal was retained, by the same rule applied to probes. Panel coverage ranged from seventy-four to ninety-seven of ninety-nine genes across cohorts (Table 1; Supplementary Figure S1). Two different gene sets are therefore used below, and the distinction matters for reading Table 2. Gene-level cross-organ comparisons were restricted to the seventy-one genes measured in all three primary cohorts. Module scores, by contrast, were computed from the genes of that module measured in the cohort concerned, so the number of genes contributing to a module score differs between cohorts; those numbers are given in the Table 2 footnote and in Supplementary Table S3. The response of all ninety-nine panel genes in all eight cohorts, not only the seventy-one held in common, is given in Supplementary Figure S2.

### 2.4. Statistical Analysis

The three primary cohorts sit on different platforms in different centres, so expression values were never pooled and no cross-cohort batch correction was applied. Each patient contributed a within-person difference, the post-reperfusion value minus the pre-reperfusion value for that same graft, computed entirely inside its own dataset. Platform and centre effects are shared by the two members of a pair and therefore cancel within the difference. Only these differences were compared between organs.

Within each cohort, each gene and each module score was tested against no change by the paired Wilcoxon signed-rank test, with false discovery rate controlled across the panel by the Benjamini–Hochberg procedure [37]. Module scores were the mean difference across the module’s genes for that patient. Effect size is reported as the median within-person change in log_2_ units and as the standardised difference.

Two metrics were carried throughout because they fail in different ways. The median log_2_ change is interpretable but depends on the dynamic range of the measurement platform. The proportion of patients whose within-person change is positive does not, and it is therefore the metric used wherever an array cohort is compared with the RNA-sequencing cohort. Heterogeneity between organs was tested on the patient-level differences by the Kruskal–Wallis test for magnitude and by the chi-square test on the proportion of patients moving upwards for direction. Confidence intervals for medians are bootstrap percentile intervals from ten thousand resamples. All computation was carried out in Python 3.11.10; numerical work relied on NumPy and pandas, statistical testing on SciPy 1.17.1 and statsmodels 0.15.0, and all figures were drawn with Matplotlib 3.11.1. *p* values are given exactly rather than as thresholds.

### 2.5. Meta-Analysis Across Cohorts

For the two panel-level findings common to all eight cohorts (the NFE2L2 axis and the metallothioneins), the within-person module score was summarised per cohort as a one-sample standardised effect size (Cohen’s *d_z_*, the mean divided by the standard deviation of the within-person differences), with variance approximated as 1/*n* + *d_z_*^2^/(2*n*). These eight cohort-level effect sizes were combined by random-effects meta-analysis with the DerSimonian–Laird estimator of between-cohort variance (statsmodels ‘combine_effects‘), giving a pooled effect, its 95% confidence interval, Cochran’s *Q* and *I*^2^ [38].

This pooling step is parametric, whereas every gene- and module-level test in this paper is the paired Wilcoxon signed-rank test, and the reason for the change deserves statement. The within-cohort tests are applied to individual genes, where a small number of patients with extreme within-person differences can dominate a mean; a rank test is the safer choice there. The pooling step operates not on patients but on eight cohort-level summaries, and standard random-effects meta-analysis requires effect sizes with estimated variances rather than ranks. Because Cohen’s *d_z_* and the DerSimonian–Laird estimator both assume approximately normally distributed effect estimates, and two of the eight contributing cohorts are small (GSE12720, *n* = 21; GSE15480, *n* = 12), the pooled estimate was re-computed with those two cohorts excluded, singly and together, and also with every cohort of fewer than twenty-five patients excluded. A distribution-free alternative was computed alongside it: the proportion of all patients in all eight cohorts whose within-person module score was positive, tested against one half by the exact binomial test. Results of both checks are reported in Section 3.5.

Because organ is the comparison of interest, Cochran’s *Q* from the eight-cohort pooling was partitioned into a between-organ and a within-organ component, the first on two degrees of freedom and the second on five, each tested against the chi-square distribution. The ratio of the between-organ component to the total is reported as the proportion of the heterogeneity attributable to organ, and the within-organ component asks whether cohorts representing the same organ differ from one another. The cohort effect sizes were also regressed on the interval from reperfusion to biopsy by weighted least squares, with weights the inverse of the within-cohort variance plus the between-cohort variance estimated above. Seven of the eight cohorts contribute to that regression; GSE151648 is excluded because its source publication does not state the interval.

### 2.6. Leave-One-Cohort-Out Sensitivity

Because each organ in the primary comparison is represented by a single cohort, the cross-organ heterogeneity claim was re-tested after substituting, one at a time, each replication cohort for its matched primary cohort (the living-donor kidney arm for GSE43974 deceased; GSE12720, GSE15480 or GSE23649 in turn for GSE151648; GSE145989 for GSE268041), holding the other two organs at their primary cohort. This asks whether the qualitative conclusion—both modules rising in all three organs, with the panel heterogeneous across organs—depends on which available cohort stands for each organ.

### 2.7. Clinical and Donor Covariate Stratification

Five of the eight cohorts carry a clinical or donor covariate alongside the paired biopsies in their deposited metadata: IRI status in GSE151648 (23 IRI-positive, 17 IRI-negative, as classified by the depositing investigators); delayed graft function in GSE43974 deceased donors (45 with, 51 without) and donor type within the same cohort (67 donation after brain death, 29 donation after circulatory death); early allograft dysfunction status in the donation-after-brain-death arm of GSE23649 (8 with, 8 without); ischaemic-preconditioning trial arm in GSE15480 (6 preconditioned, 6 not); and donor HCV serostatus in GSE12720 (10 positive, 11 negative). IRI status was extracted from the GSE151648 series matrix and donor HCV serostatus from the GSE12720 series matrix; the remaining covariates were already captured in cohort processing. For each covariate, the within-person module score for the NFE2L2 axis and the metallothioneins was compared between arms by the two-sided Mann–Whitney U test, with Benjamini–Hochberg correction across the twelve resulting tests.

The early allograft dysfunction comparison uses sixteen of the thirty-three paired GSE23649 patients, and the reason is a feature of that deposit rather than missing data. The depositing investigators classified their thirty-three patients by donor origin into four groups: nine living donors, eight donors after circulatory death, eight donors after brain death in whom the recipient developed early allograft dysfunction, and eight donors after brain death in whom the recipient did not. Early allograft dysfunction status is therefore recorded only within the donation-after-brain-death stratum, and the seventeen patients without such a label are the living-donor and circulatory-death groups, for whom the depositors recorded donor type instead. The comparison is accordingly a comparison of eight against eight within one donor stratum, not a comparison from which half a cohort has been dropped at random. The seventeen excluded patients differ systematically in donor origin, so this test is informative within the donation-after-brain-death stratum and carries no information about any association between early allograft dysfunction and induction in living or circulatory-death donors. Because this is one of the twelve tests in the corrected family, the effect of removing it was also computed and is reported in Section 3.6.

Transplant-related procedural variables were sought in every deposit and are largely absent. None of the eight cohorts records cold ischaemia time, warm ischaemia time or a preservation-solution field at sample level; the two lung cohorts deposit a gene-level matrix with no sample characteristics at all. What is recorded is donor type (GSE43974, GSE23649, GSE12720), the ischaemic-preconditioning arm (GSE15480), IRI status (GSE151648), delayed graft function and donor HCV serostatus, and the interval from reperfusion to biopsy, which is a property of each cohort rather than of each patient and is therefore handled by the meta-regression of Section 2.5 and the matched-interval contrast of Section 3.3. Donor type was accordingly tested within every cohort that records it, by the Mann–Whitney U test for two groups and the Kruskal–Wallis test for three, giving four tests additional to the twelve above. Because all sixteen are tests of the same two module scores against a covariate, the Benjamini–Hochberg correction is applied across all sixteen; the four new tests corrected on their own are also reported, so that the effect of the choice is visible.

### 2.8. Within-Organ Gene Co-Regulation

As a check on whether the ten modules reflect genuinely coordinated regulation rather than an arbitrary grouping, the Spearman correlation of the within-person change was computed between every pair of panel genes measured in each primary cohort, and genes were ordered by average-linkage hierarchical clustering on one minus that correlation, independently in each organ.

### 2.9. Single-Cell Datasets and Cell-Type Attribution

Bulk biopsy expression is a weighted average over the cell types present, so it cannot say which cells carry a response. Two public single-cell datasets of the same clinical contrast were therefore analysed alongside the bulk cohorts. GSE220797 [39] provides droplet single-cell RNA sequencing of lung biopsies from six recipients, each sampled at the end of cold ischaemia and 2 h after reperfusion—the same centre, protocol and sampling interval as the bulk primary lung cohort GSE268041, and paired within patient in the same way. GSE171539 [40] provides the corresponding liver contrast (end of preservation versus 2 h post-reperfusion) but in a single donor, and is therefore used only as supporting cell-type attribution and never as evidence of effect size. A third liver series (GSE189539) was examined and could not be used: the only matrix deposited in GEO is a merged counts table whose column names carry no sample or timepoint identifier, so its paired structure is not recoverable from the deposited data.

Processing used Scanpy 1.11 [41]. Cells were retained with 200–6000 detected genes and under 20% mitochondrial counts; counts were library-size normalised to 10,000 and log1p transformed; 2000 highly variable genes were selected (batch-aware by patient in the lung dataset), scaled, and reduced to 50 principal components. Lung patients were integrated with Harmony [42] before neighbourhood graph construction, and clusters were obtained by Leiden community detection at resolution 1.0. Cell identity was assigned from canonical lineage markers only, by scoring each cluster for each marker set and taking the best-scoring lineage; clusters whose best marker score fell below a fixed threshold of 0.05 were left as unassigned rather than forced into a label, and no reference-based label transfer was used. That threshold left no cluster unassigned in the lung dataset, where all thirty-eight Leiden clusters scored above it, so no lung cell was excluded on this basis. In the liver dataset it left one cluster of thirty-four unassigned, with a best marker score of 0.012, accounting for 136 of the 8802 cells in the paired contrast (1.5%). A cell type was tested only if at least twenty cells of that type were recovered at each of the two timepoints; the populations this excluded are named in Section 3.8.

Two further quantities were computed because a per-cell test treats cells, not patients, as the unit of analysis. First, for each cell type the smallest standardised difference detectable with 80% power at a two-sided alpha of 0.05 was obtained from its two cell counts, so that a null result can be read against what that cell type could have detected. Second, in the lung dataset, where six recipients each contribute both timepoints, the within-person change in each module was computed separately for every recipient and cell type, and the number of recipients moving upwards was tested against chance by the exact sign test. This asks whether a result significant across pooled cells is also consistent across individuals. The liver dataset comes from one donor and cannot answer that question, which is itself reported. The same a priori 99-gene panel used for the bulk analysis was then scored per cell with Scanpy’s expression-matched control-gene method, and within each cell type the post-reperfusion cells were compared with the pre-reperfusion cells of that same cell type by the Mann–Whitney U test with Benjamini–Hochberg correction across all cell type by module tests in that dataset. The catalase module contains a single gene and cannot be scored by this method; it is omitted from the single-cell analysis and reported from the bulk cohorts only.

## 3. Results

### 3.1. The Reperfusion Response Is Not a General Rise in Antioxidant Gene Expression

Within-person changes were computed for one hundred and ninety recipient pairs in the three primary cohorts and for a further one hundred and sixty-three pairs in the replication cohorts. In the primary cohorts, twenty-seven of ninety-seven panel genes changed significantly in the kidney, fifty-nine of seventy-four in the liver and sixty-seven of ninety-three in the lung after Benjamini–Hochberg control. In every organ, significant decreases outnumbered significant increases: nineteen against eight in the kidney, thirty-nine against twenty in the liver and forty against twenty-seven in the lung (Supplementary Figure S3).

**Figure 1:**
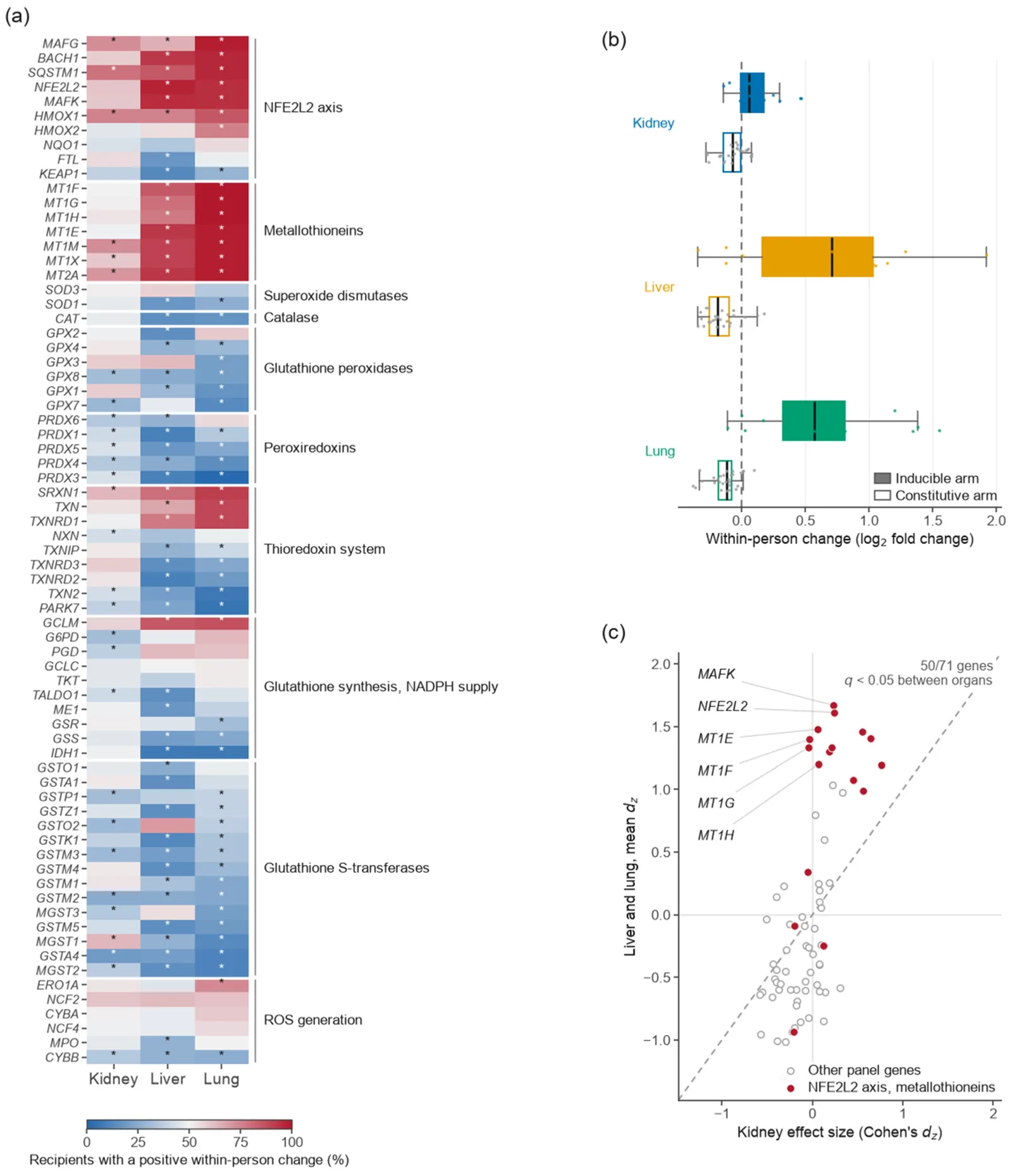
The antioxidant response to graft reperfusion in the three primary cohorts. (**a**) Within-person change in each panel gene. Colour encodes the percentage of recipients whose post-reperfusion value exceeded their own pre-reperfusion value, centred on 50%. Genes are grouped by functional module and ordered within module by the lung value. Asterisks in (**a**) mark genes significant at a Benjamini–Hochberg false discovery rate below 0.05. (**b**) Magnitude of the same change, as a log_2_ fold change, for the inducible arm (NFE2L2 axis and metallothioneins, 17 genes) and the constitutive arm (catalase, glutathione peroxidases, peroxiredoxins and glutathione S-transferases, 27 genes). Boxes span the interquartile range with the median marked and whiskers reaching 1.5 times that range; each point is one gene. (**c**) Standardised within-person effect size (Cohen’s *d_z_*) in kidney against the mean of liver and lung, one point per gene, with the dashed line marking equality. Genes of the NFE2L2 axis and the metallothioneins are drawn as filled circles and all remaining panel genes as open circles; the six genes lying furthest above equality are named. Fifty of the seventy-one genes differ between organs at a Benjamini–Hochberg false discovery rate below 0.05. Kidney *n* = 96 pairs, liver *n* = 40, lung *n* = 54; 71 genes measured in all three cohorts.

**Figure 2:**
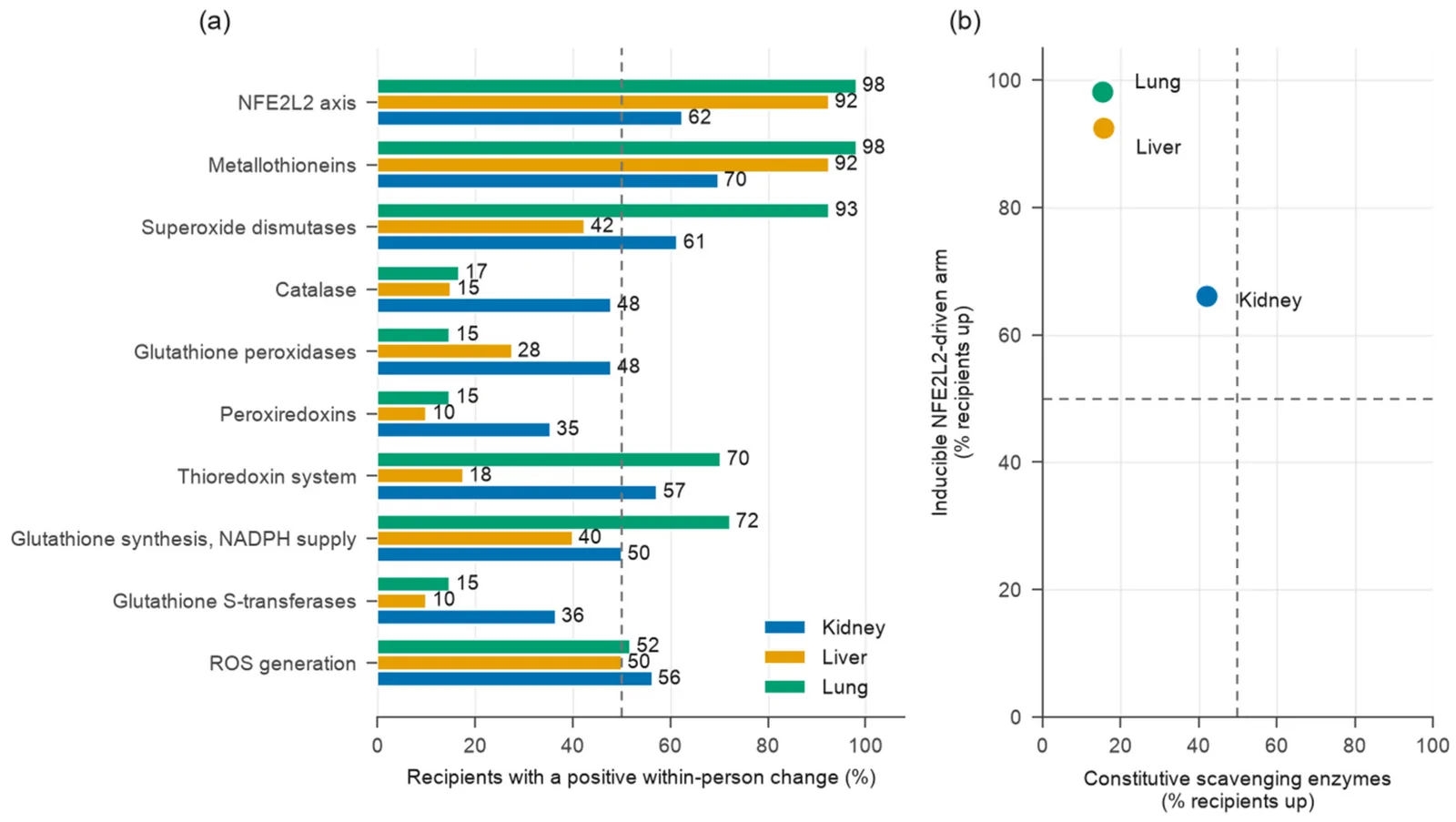
Module-level reperfusion response by organ. (**a**) Percentage of recipients with a positive within-person change for each of the ten modules; the dashed line marks the 50% null. (**b**) The inducible arm (NFE2L2 axis and metallothioneins) plotted against the constitutive arm (catalase, glutathione peroxidases, peroxiredoxins and glutathione S-transferases). All three organs lie in the upper-left quadrant, and differ in distance from the origin.

The direction of change separated by module rather than by organ (Figure 1 and Figure 2, Table 2). Transcripts of the constitutive scavenging enzymes fell. Catalase decreased in the liver and lung, rising in only 15.0% and 16.7% of recipients, respectively (*p* = 2.674 × 10^−6^ and *p* = 1.803 × 10^−6^), and did not change in kidney (47.9%, *p* = 0.169). Peroxiredoxins fell in all three organs, most strongly in lung, where *PRDX3* rose in 2% of recipients (*p* = 3.008 × 10^−10^). Glutathione peroxidases and glutathione S-transferases fell in the liver and lung and fell modestly in the kidney.

Increases were confined to the inducible arm. The NFE2L2 axis module and the metallothioneins rose in all three organs, and these were the only modules to do so. Haem oxygenase 1 rose in the kidney by a median of 0.46 log_2_ units in 75% of donors (*p* = 2.724 × 10^−7^), in the liver by 0.71 in 75% (*p* = 1.040 × 10^−5^) and in the lung by 1.38 in 85% (*p* = 1.293 × 10^−8^). *MT2A* rose in 71%, 92% and 98% of recipients, respectively (*p* = 5.982 × 10^−7^, *p* = 1.044 × 10^−8^ and *p* = 1.720 × 10^−10^).

Transcripts of the genes generating reactive oxygen species did not change in any organ: 56.2% of kidney, 50.0% of liver and 51.9% of lung recipients moved upwards, none significantly, and the module showed no heterogeneity between organs (*q* = 0.761). This is a result about transcript abundance only. Oxidant production is regulated post-transcriptionally and through enzyme activity, substrate availability and mitochondrial metabolism, so the absence of transcriptional change in these genes is not evidence that oxidant generation itself was unchanged during reperfusion.

The same distinction can be drawn within the inducible arm itself, because NFE2L2 is regulated by stabilisation of existing protein rather than by its own transcription (Supplementary Table S13). The transcripts of *NFE2L2* and its repressor *KEAP1* changed by a median of 0.19 log_2_ units in liver and 0.11 in lung, and both were significant in those two organs; the transcripts of eight NFE2L2 target genes changed by a median of 0.51 in the lung, 0.21 in the liver and −0.002 in the kidney, reaching significance in six, four and three of them, respectively. In the kidney, therefore, the sensors moved while the targets did not.

### 3.2. Induction Is Graded by Organ, and Specific Effectors Diverge

Of the seventy-one genes measured in all three primary cohorts, fifty showed heterogeneity between organs in the magnitude of the within-person change and fifty-three in its direction after Benjamini–Hochberg control (Supplementary Figure S4). At the module level, nine of ten modules were heterogeneous; the exception was reactive oxygen species generation (Table 2).

The heterogeneity took the form of a gradient in the completeness of induction rather than presence against absence. The NFE2L2 axis module rose in 98.1% of lung recipients, 92.5% of liver recipients and 62.5% of kidney donors (heterogeneity *q* = 1.112 × 10^−6^), and the metallothioneins in 98.1%, 92.5% and 69.8% (*q* = 2.797 × 10^−5^). The kidney showed the smallest proportion of the three, but the increase in the kidney was still significant for both modules (*p* = 5.767 × 10^−4^ and *p* = 1.612 × 10^−3^).

Individual effectors separated the organs more sharply than the modules did (Table 3). *GCLM*, the modifier subunit of glutamate-cysteine ligase and the rate-limiting step of glutathione synthesis, rose in the liver (median 0.47, 85% of recipients, *p* = 4.766 × 10^−7^) and in the lung (0.80, 87%, *p* = 3.585 × 10^−9^) but not in the kidney (0.07, 57%, *p* = 0.075). *TXNRD1* rose in the liver (0.21, 78%, *p* = 3.168 × 10^−4^) and lung (0.47, 89%, *p* = 3.324 × 10^−8^) but not in the kidney (−0.02, 49%, *p* = 0.795). The thioredoxin system as a whole diverged in direction: it rose in the lung (70.4%, *p* = 7.974 × 10^−4^), fell in the liver (17.5%, *p* = 3.490 × 10^−7^) and did not move in the kidney (57.3%, *p* = 0.551).

Plotted as inducible arm against constitutive arm, all three organs occupy the same quadrant—inducible up, constitutive down—and differ in the magnitude of the displacement, with kidney closest to the origin (Figure 2).

### 3.3. The Organ Gradient Is Partly, but Not Wholly, a Sampling Artefact

Two features of the assembled data could produce an apparent organ difference without any organ biology, and both were quantified rather than assumed.

Platform dynamic range is the larger of the two. Within liver alone, the three array cohorts gave a median NFE2L2 axis change of 0.094 log_2_ units against 0.429 in the RNA-sequencing cohort (*p* = 4.142 × 10^−8^), and a metallothionein change of 0.186 against 1.012 (*p* = 2.041 × 10^−8^). On the scale-free metric this narrowed to 78.8% against 92.5% of recipients for the NFE2L2 axis, and to 81.8% against 92.5% for the metallothioneins. Magnitudes in log_2_ units are therefore not comparable between an array cohort and a sequencing cohort, and all cross-organ statements above rest on the proportion metric.

**Figure 3:**
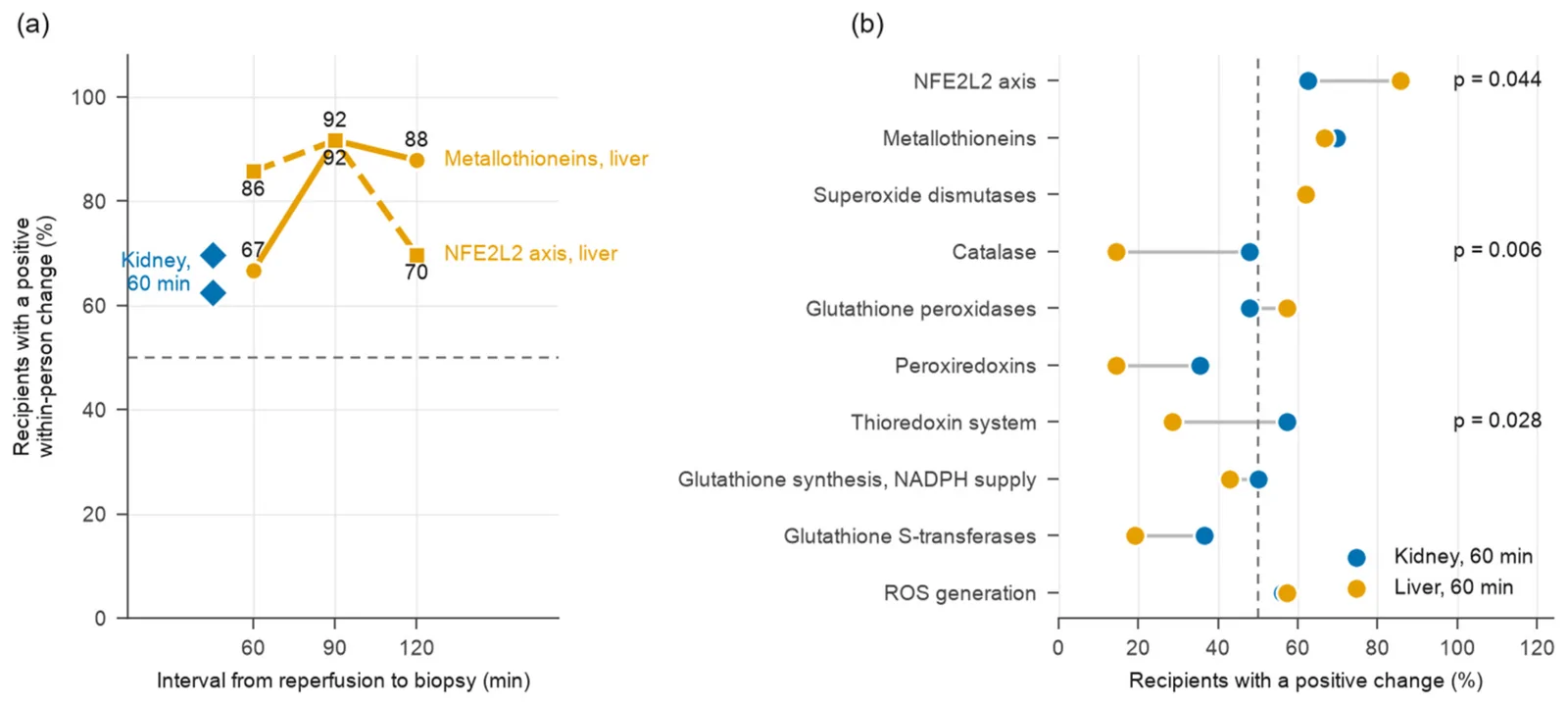
Separating organ from sampling interval. (**a**) Liver response against the interval from reperfusion to biopsy, using the three liver array cohorts sampled at 60, 90 and 120 min, with the kidney cohort shown at its own 60 min interval. (**b**) Kidney against liver at a matched interval of 60 min, module by module, the grey line joining the two organs within a module; *p* values are two-sided Fisher exact tests on the proportion of recipients moving upwards, shown where below 0.05. Where the two markers overlap, the kidney and liver values differ by less than one plotting symbol; the values themselves are given in Table 2.

The interval between reperfusion and biopsy is the second. The kidney was sampled at sixty minutes, the lung at one hundred and twenty, and the liver primary cohort at an interval its source publication does not state. Across the three liver array cohorts sampled at sixty, ninety and one hundred and twenty minutes, the metallothionein response rose with interval (Spearman ρ = 0.328, *p* = 0.007159) while the NFE2L2 axis response did not (ρ = −0.384, *p* = 0.001448), so the interval matters and matters differently by module (Figure 3a).

The one contrast in which interval is matched is kidney against liver at sixty minutes. There the organ difference persists for some modules and disappears for others (Figure 3b). The NFE2L2 axis remained higher in the liver (85.7% against 62.5%, *p* = 0.044), as did catalase suppression (14.3% against 47.9%, *p* = 0.006) and the thioredoxin system (28.6% against 57.3%, *p* = 0.028). Metallothioneins and superoxide dismutases, by contrast, were indistinguishable at matched interval (66.7% against 69.8%, *p* = 0.798; 61.9% against 61.5%, *p* = 1.000). Part of the apparent metallothionein gradient in Table 2 is therefore attributable to sampling time, whereas the NFE2L2 gradient survives interval matching.

### 3.4. Replication

**Figure 4:**
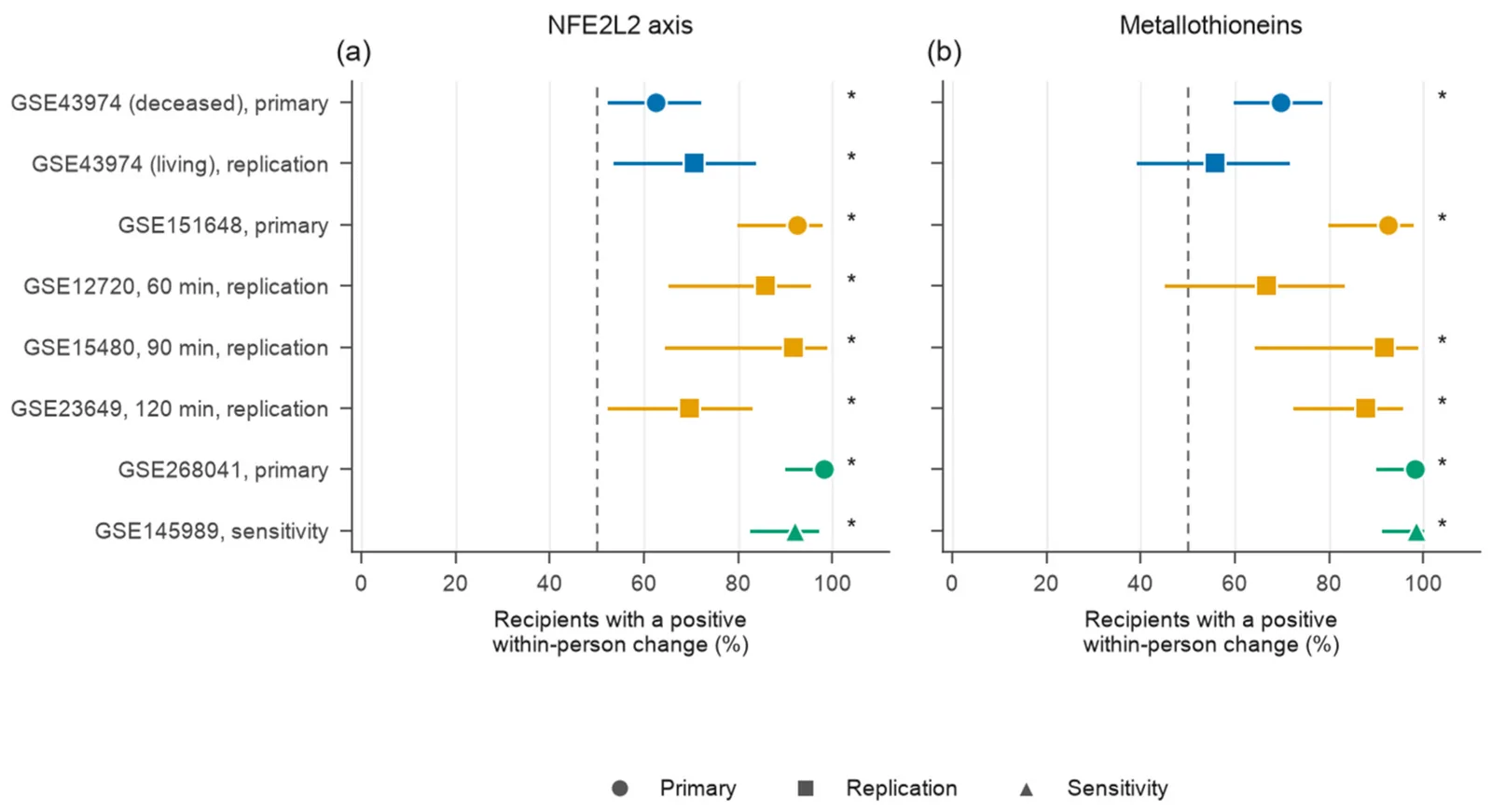
Replication across all eight cohorts. (**a**) NFE2L2 axis and (**b**) metallothionein module response in each of the eight cohorts, ordered by organ. Points are the percentage of recipients with a positive within-person change; horizontal lines are 95% Wilson confidence intervals; asterisks mark *p* < 0.05 by the paired Wilcoxon signed-rank test. Marker shape encodes the role of the cohort (primary, replication or sensitivity); colour encodes organ as in Figure 1, Figure 2 and Figure 3. GSE145989 is shown as sensitivity throughout because it shares 47 patient identifiers with GSE268041 and is not an independent replicate.

The direction of the two central findings held across all eight cohorts (Figure 4). The NFE2L2 axis module rose significantly in all eight. The metallothioneins rose in direction in all eight but reached nominal significance (*p* < 0.05) in six of eight; the two exceptions were the smallest liver replication cohort (GSE12720, *n* = 21, *p* = 0.082) and the living-donor kidney arm (*n* = 34, *p* = 0.059), both under-powered rather than discordant in direction. Constitutive scavenger modules fell or did not change in every cohort.

The living-donor arm of GSE43974, in which grafts are not exposed to donor brain death or prolonged cold ischaemia, showed the same pattern in attenuated form. Of the ninety-seven panel genes measured in that arm, six changed significantly after Benjamini–Hochberg control, and all six increased: *MT1M* (median 0.77 log_2_ units, 88.2% of donors, *q* = 7.00 × 10^−6^) and *MT2A* (0.35, 73.5%, *q* = 4.55 × 10^−2^) from the metallothionein module; *SQSTM1* (0.34, 91.2%, *q* = 7.00 × 10^−6^), *MAFG* (0.17, 79.4%, *q* = 4.06 × 10^−4^) and *HMOX1* (0.22, 64.7%, *q* = 1.13 × 10^−2^) from the NFE2L2 axis module; and *SRXN1* (0.20, 82.4%, *q* = 1.24 × 10^−3^), the sulphiredoxin of the thioredoxin system module and itself an NFE2L2 target gene. All six therefore fall within the inducible arm, and none within the constitutive scavenging modules.

### 3.5. Meta-Analysis Confirms a Moderate, Heterogeneous Pooled Effect

**Figure 5:**
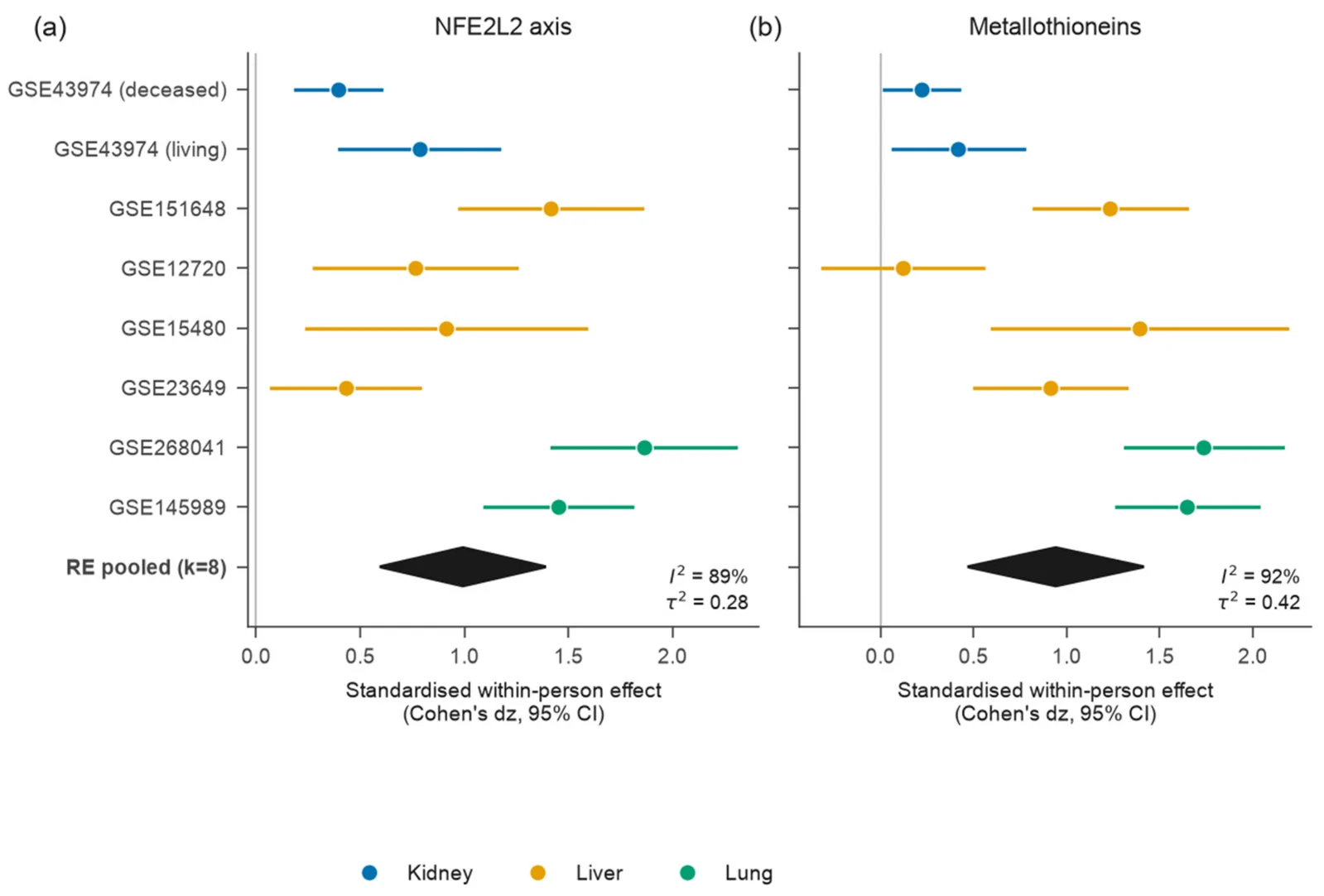
Random-effects meta-analysis of (**a**) the NFE2L2 axis and (**b**) the metallothioneins across all eight cohorts. Points and horizontal lines are the cohort-level standardised effect size (Cohen’s *d_z_*) with 95% confidence interval; colour encodes organ as in Figure 1, Figure 2, Figure 3 and Figure 4. The diamond is the DerSimonian–Laird pooled random-effects estimate with its 95% confidence interval; *I*^2^ and τ^2^ quantify between-cohort heterogeneity.

Random-effects meta-analysis of the eight cohort-level effect sizes gave a pooled standardised effect of 0.99 (95% CI 0.59–1.39) for the NFE2L2 axis module and 0.94 (95% CI 0.47–1.42) for the metallothioneins (Figure 5); both intervals exclude zero. Heterogeneity between cohorts was substantial for both (*I*^2^ = 89% and 92%, respectively, τ^2^ = 0.28 and 0.42). The pooled analysis therefore supports an overall positive response across cohorts while also demonstrating substantial between-cohort heterogeneity, and the pooled value should be read as a summary of responses of differing size rather than as an estimate of one common effect.

The robustness of that pooled estimate to the two smallest contributing cohorts was tested directly. Excluding GSE15480 (*n* = 12) moved the pooled estimate for the NFE2L2 axis module from 0.99 to 1.00; excluding GSE12720 (*n* = 21) moved it to 1.03; excluding both moved it to 1.04 (95% CI 0.55–1.54), a shift of 0.05; and excluding every cohort of fewer than twenty-five patients gave the same six-cohort result. For the metallothioneins the corresponding values were 0.89, 1.06 and 1.02 (95% CI 0.46–1.57) against 0.94 for all eight. The two small cohorts pull in opposite directions for that module, GSE15480 upwards (*d_z_* 1.39) and GSE12720 downwards (*d_z_* 0.12), so removing either alone moves the pooled estimate either way; removing both leaves it between 0.9 and 1.1. Every interval computed in these scenarios excludes zero, and the heterogeneity remains substantial throughout (*I*^2^ 89–94%). The distribution-free alternative agrees: across all 353 patients in all eight cohorts, the within-person NFE2L2 axis score was positive in 284 (80.5%, exact binomial *p* = 4.23 × 10^−32^) and the metallothionein score in 292 (82.7%, *p* = 2.85 × 10^−37^), with the cohort-level proportion ranging from 62.5% to 98.1% and from 55.9% to 98.4%, respectively. The direction of the effect therefore does not depend on the parametric pooling step, and neither does the conclusion that its size varies between cohorts.

Partitioning that heterogeneity shows where it comes from (Supplementary Table S11). For the NFE2L2 axis module, 44.64 of the total *Q* of 61.58 lies between organs (2 df, *p* = 2.03 × 10^−10^), so 72.5% of the heterogeneity is attributable to organ; for the metallothioneins the between-organ component is 71.19 of 88.48, or 80.5% (*p* = 3.48 × 10^−16^). Pooled within organ, the NFE2L2 axis module gives *d_z_* 0.56 (95% CI 0.18 to 0.93) in the kidney, 0.87 (0.40 to 1.34) in the liver and 1.63 (1.23 to 2.04) in the lung, reproducing the gradient on the effect-size scale as well as on the proportion scale. The remaining within-organ component is not zero: 16.94 on 5 df (*p* = 0.0046) for the NFE2L2 axis module and 17.29 (*p* = 0.0040) for the metallothioneins. It is concentrated in the liver, the only organ whose cohorts differ significantly among themselves (NFE2L2 axis *I*^2^ = 74.7%, *p* = 0.0079; metallothioneins *I*^2^ = 81.6%, *p* = 0.0010), whereas the two kidney cohorts and the two lung cohorts do not differ (all *p* > 0.08). Liver is also the organ represented by four cohorts spanning two measurement platforms and three sampling intervals, which Section 3.3 quantifies directly.

Consistent with that, regressing the cohort effect sizes on the interval from reperfusion to biopsy separates the two modules. The metallothionein effect size rises with interval (0.020 *d_z_* per minute, 95% CI 0.002 to 0.038, *p* = 0.032), while the NFE2L2 axis module does not (0.010 per minute, *p* = 0.196); residual heterogeneity after allowing for interval is small in both (*p* = 0.60 and 0.92). This is the same dissociation found in the matched-interval contrast of Section 3.3, reached by a different route.

### 3.6. The Organ-Level Conclusions Survive Substitution of Cohorts

**Figure 6:**
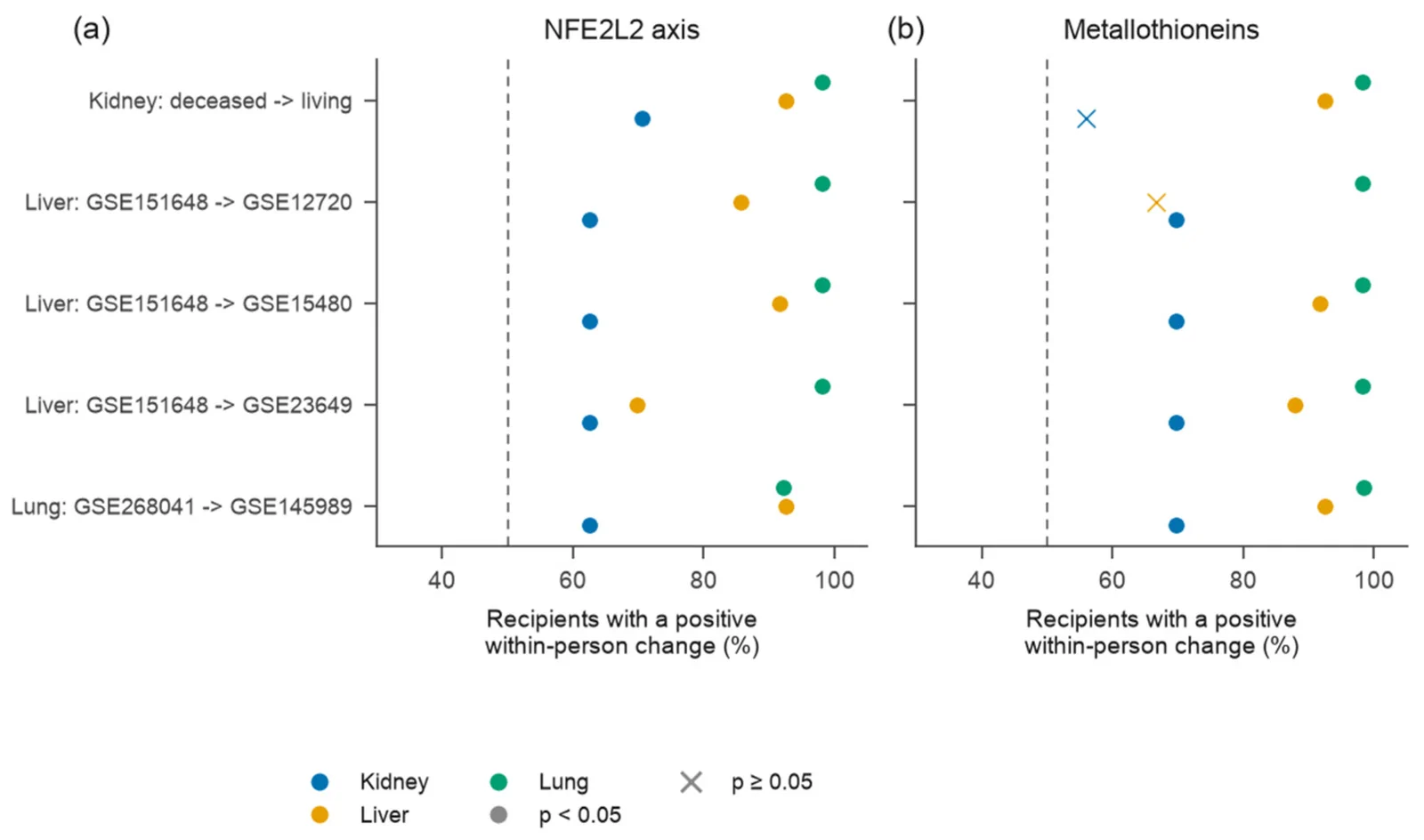
Leave-one-cohort-out sensitivity of the primary cross-organ comparison for (**a**) the NFE2L2 axis and (**b**) the metallothioneins. Each row recomputes the three-organ comparison after substituting the named replication cohort for its matched primary cohort, holding the other two organs at their primary cohort; points are the percentage of recipients with a positive within-person change in each organ, filled circles marking *p* < 0.05 and crosses marking *p* ≥ 0.05 by the paired Wilcoxon signed-rank test within that organ. In the legend, the grey circle and the grey cross indicate symbol shape only, that is significance; colour indicates organ, so no grey symbol appears in either panel.

Recomputing the three-organ heterogeneity comparison with each replication cohort standing in turn for its matched primary cohort left the qualitative pattern intact in eight of the ten swap-by-module combinations tested (Figure 6): the NFE2L2 axis remained significant in all three organs under every substitution, and the metallothioneins in all but the two substitutions that used the smallest available cohort for kidney or liver (living-donor kidney, *n* = 34; GSE12720 liver, *n* = 21)—the same two cohorts that were under-powered, not discordant, in the replication analysis above. The organ gradient and the conclusion that both modules are induced across kidney, liver and lung are therefore not an artefact of which single cohort happens to represent each organ.

### 3.7. Induction Is Not Explained by the Clinical or Donor Covariates Available

**Figure 7:**
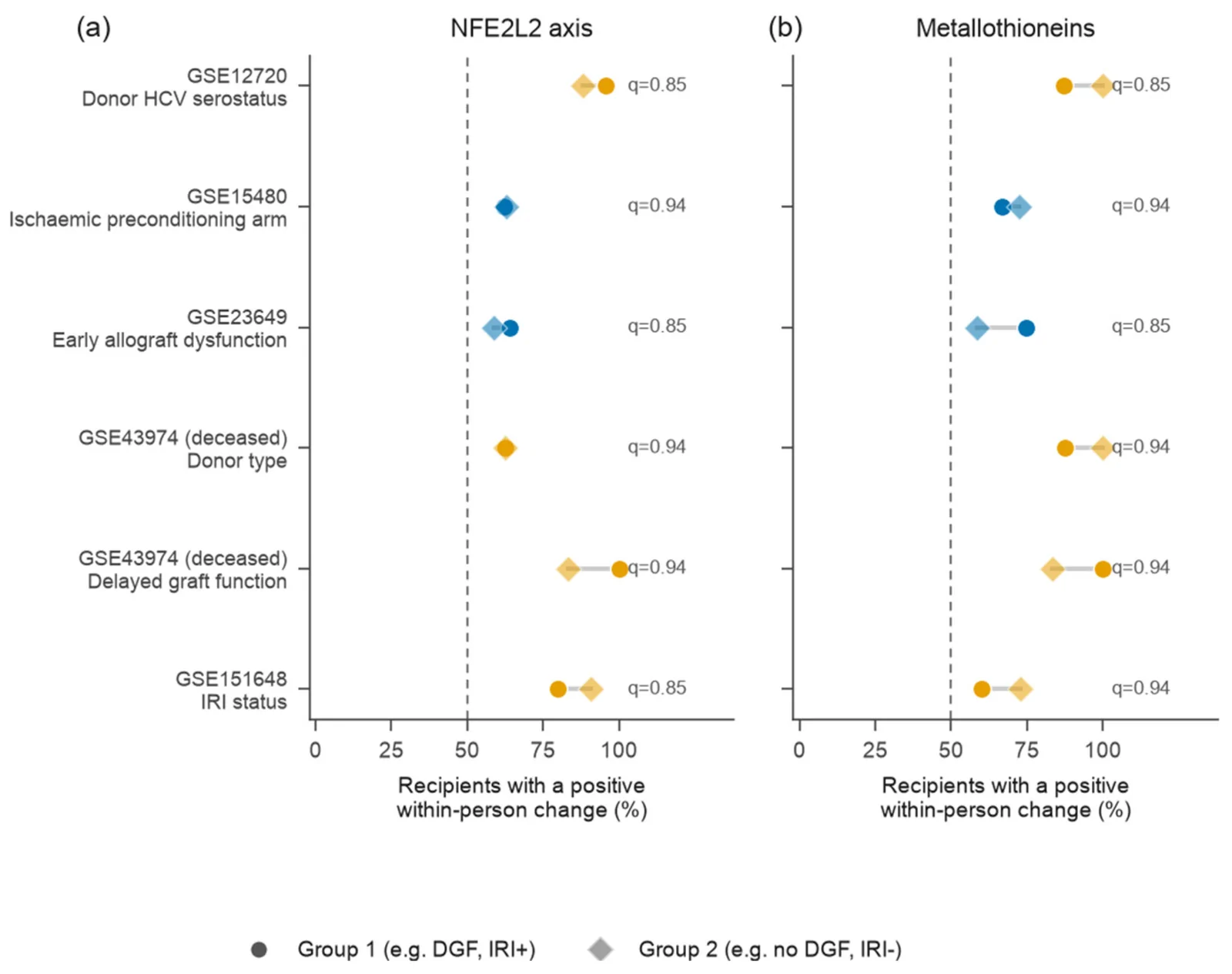
Robustness of induction to six clinical and donor covariates, drawn from the five cohorts in which they were recorded. (**a**) NFE2L2 axis and (**b**) metallothionein module response, plotted as the percentage of recipients with a positive within-person change in each arm of each covariate (circle, first-named group; diamond, second-named group; connecting line for visual reference only). Colour encodes organ as in Figure 1, Figure 2, Figure 3 and Figure 4. q values are Benjamini–Hochberg adjusted across all twelve tests (two modules x six covariates).

Five cohorts carried a clinical or donor covariate alongside the paired biopsies. Comparing the within-person module score between the two arms of each covariate gave no evidence of association: IRI-positive and IRI-negative liver recipients induced the NFE2L2 axis and the metallothioneins to a similar degree (GSE151648, both *p* > 0.18); delayed graft function was not associated with induction in kidney (both *p* > 0.6); nor was donor type (donation after brain death versus circulatory death, both *p* > 0.15), early allograft dysfunction (both *p* > 0.6), ischaemic-preconditioning trial arm (both *p* > 0.8) or donor HCV serostatus (both *p* > 0.27) (Figure 7, Supplementary Figure S6). None of the twelve tests reached Benjamini–Hochberg significance, the smallest adjusted value being *q* = 0.85.

Two features of this analysis are stated explicitly. The early allograft dysfunction comparison uses sixteen of the thirty-three paired GSE23649 patients, because the depositing investigators recorded that status only within the donation-after-brain-death stratum (Section 2.7); the remaining seventeen are the nine living-donor and eight circulatory-death patients, and this test carries no information about those two groups. Removing its two tests from the corrected family leaves ten tests whose smallest adjusted value is *q* = 0.70 rather than 0.85, so the conclusion that no covariate reached significance does not depend on their inclusion, although the numerical bound does. Several of these comparisons are also small, with as few as six patients per arm, and cannot exclude a true association. What they show is that the induction documented above is not obviously confounded by, or a proxy for, these five covariates: it occurs in donors and recipients on both sides of each one.

Donor type was then tested in all three cohorts that record it (Supplementary Table S12). In the kidney primary cohort the recomputation reproduced the published values exactly (donation after brain death against donation after circulatory death, *p* = 0.346 and *p* = 0.158). In GSE12720 the two donor types were indistinguishable for both modules (*p* = 0.697 and *p* = 0.269). In GSE23649, which records four donor groups, the NFE2L2 axis module differed between them at a nominal *p* = 0.0083: the within-person increase was largest in living donors (median 0.177 log_2_ units, 89% of patients up, *n* = 9), intermediate in donation after brain death (0.049, 62%, *n* = 16) and smallest in donation after circulatory death (0.002, 62%, *n* = 8); the metallothioneins did not differ (*p* = 0.195). This nominal association does not survive correction across the sixteen covariate tests (*q* = 0.133), although it would if the four new tests were corrected alone (*q* = 0.033). We therefore report it as a signal rather than a finding, and note that it is directionally consistent with the living-donor kidney arm of GSE43974, in which the same programme was the only part of the panel to change (Section 3.4). The module rose in the majority of patients in every donor group, so the result qualifies the magnitude of induction rather than its presence.

### 3.8. Structural and Resident Cells, Not Recruited Myeloid Cells, Carry the Induction

**Figure 8:**
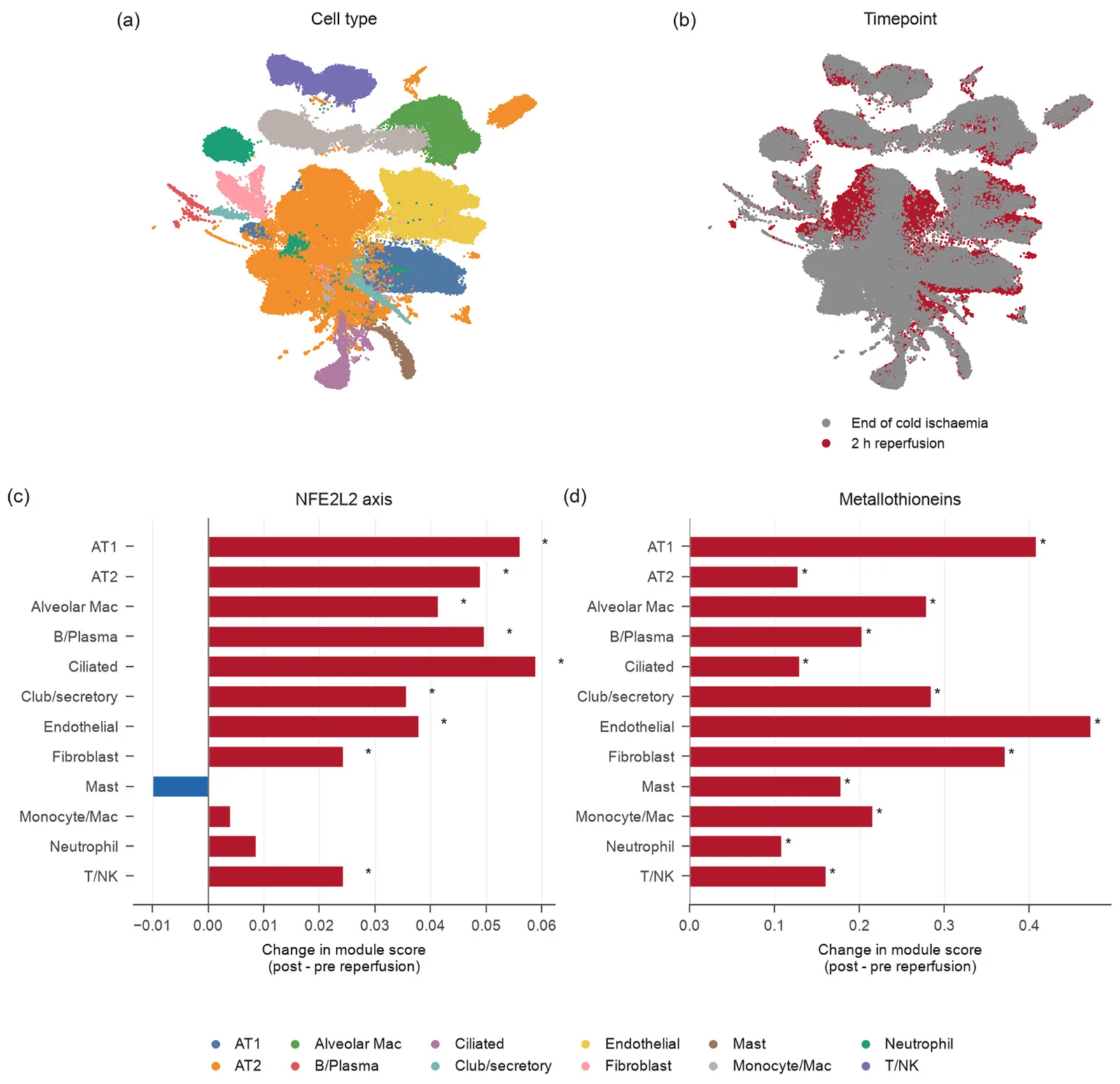
Cell-type attribution of the induction in lung, from single-cell RNA sequencing of six recipients each sampled at the end of cold ischaemia and 2 h after reperfusion (GSE220797; 117,549 cells after quality control). (**a**) Uniform manifold approximation and projection (UMAP) coloured by cell type, assigned from canonical lineage markers. (**b**) The same embedding coloured by timepoint. (**c**) Change in the NFE2L2 axis module score and (**d**) in the metallothionein module score between post- and pre-reperfusion cells of the same cell type; bars are red where the change is positive and blue where negative, and asterisks mark a Benjamini–Hochberg false discovery rate below 0.05 across all cell type by module tests in this dataset. The metallothioneins rise in every cell type; the NFE2L2 axis rises in nine of twelve but not in monocytes and monocyte-derived macrophages, neutrophils or mast cells.

Bulk biopsy expression averages over whatever cells are present, so it cannot distinguish a programme induced broadly across the graft from one confined to a few cell types, nor separate induction within cells from a change in which cells are there. Single-cell data of the same clinical contrast address this. In the lung dataset, 117,549 cells passed quality control from six recipients, each contributing both an end-of-cold-ischaemia and a 2 h post-reperfusion biopsy (58,707 and 58,842 cells, respectively), and clustered into twelve cell types assigned by canonical markers (Figure 8a,b; Supplementary Figure S7a). This count is larger than the 108,613 cells reported by the depositing investigators because the quality-control criteria of Section 2.9 were applied here to the deposited barcode matrices, which contain 134,257 barcodes in total, rather than adopting the cell set used in the source publication.

The metallothionein response was universal but strongly graded: it rose significantly in all twelve cell types, most in endothelium (change in module score 0.47), alveolar type 1 epithelium (0.41) and fibroblasts (0.37), and least in alveolar type 2 epithelium (0.13) and neutrophils (0.11) (Figure 8d). The NFE2L2 axis, by contrast, was not universal. It rose significantly in nine of twelve cell types—most in ciliated (0.059) and alveolar type 1 (0.056) epithelium, alveolar macrophages (0.041) and endothelium (0.038)—but did not change in the three recruited or granulocytic populations: monocytes and monocyte-derived macrophages (0.004, *p* = 0.81), neutrophils (0.009, *p* = 0.28) and mast cells (−0.010, *p* = 0.30) (Figure 8c). The constitutive arms behaved as they did in bulk: peroxiredoxins fell in every cell type and significantly so in ten of twelve, and the glutathione S-transferases fell significantly in nine of twelve, rising significantly only in B and plasma cells. Superoxide dismutases were the exception in both layers, rising significantly in seven of twelve cell types, which matches their behaviour in the bulk lung cohort (Table 2).

The same split appeared in the liver dataset, which comes from a single donor and is therefore reported only as supporting cell-type attribution (Supplementary Figure S8). Of the three timepoints deposited for that donor, the two forming the paired contrast used here, the end of preservation and 2 h after portal reperfusion, retained 5100 and 3702 cells, respectively, after quality control, 8802 cells in total. Eight cell types were assigned, and 136 cells (1.5%) were left unassigned. Six of the eight met the minimum of twenty cells at each timepoint and were tested: T and NK cells (2389 cells before, 1205 after), B and plasma cells (750, 453), monocytes (682, 783), endothelium (592, 669), Kupffer cells and macrophages (456, 507), and stellate cells (35, 31). Hepatocytes (55, 17) and cholangiocytes (13, 29) did not meet that minimum and were not tested, so no statement is made about them. Among the six tested, metallothioneins rose in stellate cells, endothelium and Kupffer cells, and the NFE2L2 axis module rose significantly in four of six; monocytes were again the population that did not increase the module (*p* = 0.61) and was the only one whose metallothionein score fell.

The unit of analysis in these tests is the cell, and two checks bound what that supports (Supplementary Tables S14 and S15). On power, the cell counts are ample for the populations tested: the smallest standardised difference detectable with 80% power is 0.03 to 0.20 across the twelve lung cell types and 0.10 to 0.18 for five of the six liver cell types, so the absence of an NFE2L2 axis response in monocytes, neutrophils and mast cells is informative rather than merely unpowered. The exception is liver stellate cells, where 35 and 31 cells give a detectable difference in only 0.69, so the metallothionein increase reported there is the one liver result that rests on a cell count too small to interpret as an estimate.

On consistency between individuals, the lung result is weaker than the pooled-cell *p* values alone would suggest. Taking each of the six recipients separately, the NFE2L2 axis module rose in all six only in alveolar type 1 epithelium (sign test *p* = 0.031); it rose in five of six in alveolar macrophages and endothelium, and in four of six in most remaining types, so no other cell type reaches significance at the level of individuals. The direction of the per-recipient medians agrees with the pooled analysis in eleven of twelve cell types, mast cells being the exception. The cell-type attribution should therefore be read as the average behaviour of these six grafts rather than as a result established recipient by recipient, and the liver dataset, from one donor, cannot be assessed this way at all.

Two observations follow, within those limits. First, the available single-cell data support the involvement of structural and resident cell populations—epithelium, endothelium, fibroblasts and resident macrophages—rather than of the inflammatory cells arriving with reperfusion. Second, the module did not increase in monocytes or neutrophils in either dataset, which is not the pattern expected if the bulk signal were generated by an influx of such cells.

### 3.9. The Ten Modules Are Also Coherent at the Level of Individual Genes

Within each primary cohort, genes assigned to the same module by database annotation tended to cluster together when genes were ordered purely by the correlation of their within-person change (Supplementary Figure S5), most visibly for the metallothioneins and the NFE2L2 axis in all three organs. This is an internal check rather than a new finding: the modules used throughout were defined from literature and database records before any expression data were seen (Methods), and this analysis asks only whether that a priori grouping also holds together empirically, which it does.

## 4. Discussion

The first of three findings has bearing on why systemic antioxidant supplementation has performed poorly. Supplying a constitutive scavenger adds an enzyme whose transcription the tissue is not increasing, and which in liver and lung is falling. The response observed here instead raises transcription of haem oxygenase 1, the metallothioneins, the glutathione synthesis enzymes and thioredoxin reductase: an inducible stress programme rather than more constitutive enzyme. This pattern is consistent with the broader concept of adaptive tissue reprogramming under stress, in which redox signalling is integrated with metabolic and inflammatory states and may generate distinct responses across organs and cell populations [43]. It supports a therapeutic hypothesis these data do not test: that agents amplifying NFE2L2 signalling, or supplying the substrates that programme consumes, may be better matched to the observed response than scavengers. Testing it would require an interventional study.

The second would constrain how such an agent is delivered. At sixty minutes the kidney raises the programme in fewer than two-thirds of donors against more than four-fifths of livers, and does not raise *GCLM* or *TXNRD1*, which rise in the liver and lung. A uniform systemic dose would therefore encounter three different transcriptional states. The kidney is weaker rather than unresponsive: the module does rise significantly there.

The third is a caution about mechanism. The superoxide burst at reperfusion is fast and post-translational, driven by accumulated succinate acting on complex I [4]. Nothing here contradicts that; the absence of change in NOX, DUOX and xanthine oxidoreductase transcripts is what that model predicts, because the burst does not require new transcription. The biological consequences of reperfusion-associated redox stress are also strongly dependent on the local redox environment, cellular context, and temporal dynamics of reactive signalling, rather than on transcript abundance alone [44]. The layer sampled here is the response to the burst rather than its cause, so a therapy timed to the transcriptional window would address a different event.

The same caution applies to the pathway this paper is named after. NFE2L2 is activated by stabilisation of protein already present, so activation need not raise its own transcript, and a transcriptomic measurement cannot date it. The sensor-against-target pattern of Section 3.1 is consistent with that but does not demonstrate it, and nothing here measures NFE2L2 protein, its localisation, or any enzyme activity.

That machine perfusion has produced clearer improvements than pharmacological antioxidants [12,14,15] is compatible with this reading: it alters the conditions under which the graft encounters oxygen, and allows the inducible programme time to operate before the graft must support a recipient [45,46].

The single-cell layer adds cell-type resolution. An agent directed at the inducible arm would act on epithelium, endothelium, fibroblasts and resident macrophages rather than on arriving monocytes and neutrophils, and those cells are already in the graft and accessible during preservation. Resting on six lung recipients and one liver donor, and on cell-level rather than patient-level tests, both statements are hypotheses rather than established targeting principles.

Three further analyses bear on how much of this rests on any single cohort or subgroup. Most of the pooled heterogeneity is attributable to organ; cohort substitution left the conclusion intact except where the substitute was under-powered; and induction was present on both sides of every covariate tested. None excludes associations too small to detect.

The design has several strengths. Each patient is their own control, removing the between-subject variation that dominates cross-sectional comparisons of graft tissue. The panel was fixed before any expression data were examined, pairing in the largest cohort was verified against the Supplementary File of the original publication rather than assumed from sample order, and the two confounds that could produce an apparent organ effect were measured directly.

Several limitations qualify the conclusions. The first bounds every result: this study measures transcript abundance, not protein abundance, enzyme activity or functional antioxidant capacity. No cohort reports oxidative damage markers or enzyme activities for calibration, and the analysis is restricted to the pre-specified panel. Log2 magnitudes are not comparable between array and sequencing cohorts, so cross-organ claims rest on the scale-free proportion. Reperfusion interval is confounded with organ; the one matched-interval contrast rests on twenty-one liver grafts, and the liver primary cohort’s interval is unstated. No cohort records ischaemia time or preservation solution (Section 2.7), so the procedural variables that differ most between these organs cannot be adjusted for. Lung has no independent replication cohort, because the second lung series shares 47 of its patients with the first (Figure 4). Kidney biopsies come from donors and the others from recipients, so donor state differs systematically between organs.

The single-cell layer carries three further limitations. It covers two of three organs and not the kidney. The lung dataset rests on six recipients and the liver on one donor, in which two of eight assigned cell types were too sparse to test. And its tests take the cell as the unit of analysis: only one of twelve lung cell types shows the response in all six recipients, so the attribution describes the average of these grafts rather than a per-patient result. Proportions recovered by dissociation also reflect how readily each population survives handling, so composition differences between timepoints (Supplementary Figure S7b) are not read as changes in absolute cell number.

## 5. Conclusions

In paired human kidney, liver and lung transplant biopsies, reperfusion does not raise antioxidant gene expression as a whole. Transcripts of the constitutive scavenging enzymes fall or remain unchanged, while the inducible NFE2L2-dependent antioxidant programme rises with a completeness that is graded by organ—strongest in the lung, intermediate in the liver and weakest in the kidney—and with specific effectors, notably *GCLM* and *TXNRD1*, that rise in liver and lung but not in kidney. Transcripts of the enzymes that generate reactive oxygen species do not change, which does not establish that oxidant production is unchanged. These observations are transcriptional and were obtained without testing any intervention. They provide a rationale for investigating whether antioxidant strategies directed at the inducible arm, selected by organ and timed to its response, are more effective than a single systemic antioxidant applied uniformly; they do not establish that such strategies are effective.

## Figures and Tables

**Table 1:** Characteristics of the eight human transplant biopsy cohorts.

Accession	Organ	Graft Type	Pre-Reperfusion Biopsy	Post-Reperfusion Biopsy	Paired Patients	Platform	Panel Genes Measured	Role	Ref.
GSE43974 (deceased)	Kidney	DBD and DCD donors	End of cold ischaemia	60 min after reperfusion	96	Illumina HumanHT-12 v4	97	Primary	[24]
GSE151648	Liver	Deceased donor OLT	Pre-reperfusion	Post-reperfusion, interval not stated	40	Illumina HiSeq 3000 (RNA-seq)	74	Primary	[25]
GSE268041	Lung	DBD donors	End of cold preservation	120 min after reperfusion	54	Affymetrix U133 Plus 2.0	93	Primary	[28]
GSE43974 (living)	Kidney	Living donors	Donation	60 min after reperfusion	34	Illumina HumanHT-12 v4	97	Replication	[24]
GSE12720	Liver	Living and deceased donors	Backbench cold preservation	60 min after reperfusion	21	Affymetrix U133 Plus 2.0	92	Replication	[26]
GSE15480	Liver	Deceased donors, IPC trial	End of cold storage	90 min after reperfusion	12	Affymetrix Human Gene 1.0 ST	89	Replication	[27]
GSE23649	Liver	Living, DCD and DBD donors	Before cold perfusion	120 min after portal reperfusion	33	Illumina HumanHT-12 v3	97	Replication	[30]
GSE145989	Lung	DBD donors	End of cold ischaemia	60–120 min after reperfusion	63	Affymetrix U133 Plus 2.0	90	Sensitivity	[31]

DBD, donation after brain death; DCD, donation after circulatory death; IPC, ischaemic preconditioning; OLT, orthotopic liver transplantation; RNA-seq, RNA sequencing. Panel genes measured is the number of the 99 a priori panel genes present on that platform after mapping to approved gene symbols. GSE145989 shares 47 patient identifiers with GSE268041 and is therefore reported as a sensitivity analysis, not as independent replication.

**Table 2:** Module-level reperfusion response in the three primary cohorts.

Module	Panel Genes	Kidney% Up	Kidney *p*	Liver% Up	Liver *p*	Lung% Up	Lung *p*	Heterogeneity q
NFE2L2 axis	10	62.5	5.767 × 10^−4^	92.5	1.164 × 10^−9^	98.1	1.720 × 10^−10^	1.112 × 10^−6^
Metallothioneins	12	69.8	0.002	92.5	1.582 × 10^−8^	98.1	1.720 × 10^−10^	2.797 × 10^−5^
Superoxide dismutases	3	61.5	0.004	42.5	0.826	92.6	8.528 × 10^−10^	3.641 × 10^−6^
Catalase	1	47.9	0.169	15.0	2.674 × 10^−6^	16.7	1.803 × 10^−6^	2.898 × 10^−5^
Glutathione peroxidases	8	47.9	0.720	27.5	0.002	14.8	1.107 × 10^−7^	2.229 × 10^−4^
Peroxiredoxins	6	35.4	2.653 × 10^−4^	10.0	3.875 × 10^−7^	14.8	5.430 × 10^−9^	0.001
Thioredoxin system	10	57.3	0.551	17.5	3.490 × 10^−7^	70.4	7.974 × 10^−4^	3.641 × 10^−6^
Glutathione synthesis and NADPH supply	11	50.0	0.511	40.0	0.183	72.2	1.251 × 10^−4^	0.005
Glutathione S-transferases	23	36.5	0.012	10.0	2.040 × 10^−7^	14.8	1.853 × 10^−7^	9.622 × 10^−4^
ROS generation	15	56.2	0.182	50.0	0.563	51.9	0.935	0.761

Values are the percentage of patients whose within-person change (post-reperfusion minus pre-reperfusion, same graft) was positive. *p* values are two-sided paired Wilcoxon signed-rank tests against no change. Heterogeneity q is the Benjamini–Hochberg adjusted *p* value of a chi-square test comparing the proportion of patients moving upwards across the three organs. Kidney *n* = 96 pairs, liver *n* = 40, lung *n* = 54. The Panel Genes column is the number of genes the panel assigns to that module; a module score for a given cohort is computed from those of its genes measured in that cohort, which for eight of the ten modules is fewer (kidney/liver/lung): metallothioneins 11/8/10, superoxide dismutases 3/2/3, glutathione peroxidases 8/6/7, peroxiredoxins 5/6/6, thioredoxin system 10/9/10, glutathione synthesis and NADPH supply 11/10/11, glutathione S-transferases 23/16/20, and reactive oxygen species generation 15/6/15. The NFE2L2 axis (10 genes) and catalase (1) were measured in full in all three cohorts. Panel coverage overall was 97 of 99 genes in the kidney, 74 in the liver and 93 in the lung.

**Table 3:** Individual antioxidant effectors in the three primary cohorts.

Gene	Kidney Median	Kidney% Up	Kidney *p*	Liver Median	Liver% Up	Liver *p*	Lung Median	Lung% Up	Lung *p*
*HMOX1*	+0.46	75	2.724 × 10^−7^	+0.71	75	1.040 × 10^−5^	+1.38	85	1.293 × 10^−8^
*MT2A*	+0.24	71	5.982 × 10^−7^	+1.14	92	1.044 × 10^−8^	+0.57	98	1.720 × 10^−10^
*MT1X*	+0.13	59	0.020	+1.03	90	2.823 × 10^−7^	+0.77	98	1.819 × 10^−10^
*GCLM*	+0.07	57	0.075	+0.47	85	4.766 × 10^−7^	+0.80	87	3.585 × 10^−9^
*TXNRD1*	−0.02	49	0.795	+0.21	78	3.168 × 10^−4^	+0.47	89	3.324 × 10^−8^
*SLC7A11*	+0.01	55	0.739	not measured	-	-	+0.78	81	1.538 × 10^−7^
*SOD2*	+0.25	72	3.993 × 10^−6^	not measured	-	-	+1.29	91	6.499 × 10^−10^
*TXN*	+0.07	54	0.195	+0.15	68	0.008	+0.24	89	3.324 × 10^−8^
*SRXN1*	+0.05	64	0.002	+0.46	80	4.153 × 10^−6^	+0.56	91	2.132 × 10^−8^
*NQO1*	−0.15	44	0.061	−0.12	35	0.066	+0.03	56	0.411
*CAT*	−0.03	48	0.169	−0.21	15	2.674 × 10^−6^	−0.18	17	1.803 × 10^−6^
*GPX1*	+0.08	58	0.276	−0.15	30	0.002	−0.15	17	8.250 × 10^−7^
*GPX3*	+0.07	58	0.384	+0.12	62	0.037	−0.10	20	1.769 × 10^−5^
*PRDX3*	−0.03	44	0.006	−0.17	10	6.776 × 10^−9^	−0.22	2	3.008 × 10^−10^
*GSR*	+0.03	51	0.355	−0.02	45	0.900	−0.10	31	0.002
*GSTP1*	−0.26	31	2.727 × 10^−5^	−0.19	38	0.038	−0.04	39	0.007

Median is the median within-person change in log_2_ units. Median values are comparable within a column but not between the array cohorts (kidney, lung) and the RNA sequencing cohort (liver), whose dynamic ranges differ; between-organ comparison therefore uses the percentage of patients moving upwards. *p* values are two-sided paired Wilcoxon signed-rank tests. *SLC7A11* and *SOD2* were not retained after expression filtering in the liver cohort.

## Data Availability

The datasets analysed in this study are publicly available in the NCBI Gene Expression Omnibus (GEO) repository: GSE43974 (https://www.ncbi.nlm.nih.gov/geo/query/acc.cgi?acc=GSE43974 (accessed on 20 August 2026)), GSE151648 (https://www.ncbi.nlm.nih.gov/geo/query/acc.cgi?acc=GSE151648 (accessed on 20 August 2026)), GSE268041 (https://www.ncbi.nlm.nih.gov/geo/query/acc.cgi?acc=GSE268041 (accessed on 20 August 2026)), GSE12720 (https://www.ncbi.nlm.nih.gov/geo/query/acc.cgi?acc=GSE12720 (accessed on 20 August 2026)), GSE15480 (https://www.ncbi.nlm.nih.gov/geo/query/acc.cgi?acc=GSE15480 (accessed on 20 August 2026)), GSE23649 (https://www.ncbi.nlm.nih.gov/geo/query/acc.cgi?acc=GSE23649 (accessed on 20 August 2026)) and GSE145989 (https://www.ncbi.nlm.nih.gov/geo/query/acc.cgi?acc=GSE145989 (accessed on 20 August 2026)). The single-cell datasets are GSE220797 (lung; https://www.ncbi.nlm.nih.gov/geo/query/acc.cgi?acc=GSE220797 (accessed on 20 August 2026)) and GSE171539 (liver; https://www.ncbi.nlm.nih.gov/geo/query/acc.cgi?acc=GSE171539 (accessed on 20 August 2026)). Processed panel-level and per-cell-type results generated in this study are provided in the Supplementary Materials. All analysis code and every derived table behind the figures and tables are publicly available at https://github.com/sanghyunn82-creator/reperfusion-antioxidant (accessed on 7 October 2026) under the MIT licence.

## Supplementary Materials

The following supporting information can be downloaded at: https://www.mdpi.com/article/10.3390/biom16101485/s1, Figure S1: Cohort overview—paired patients and antioxidant/ROS panel gene coverage in each of the eight cohorts; Figure S2: Within-person change for all 99 panel genes in all eight cohorts; Figure S3: Volcano plots of the within-person response in the three primary cohorts (kidney, liver, lung); Figure S4: Cross-organ heterogeneity for the 71 genes measured in all three primary cohorts; Figure S5: Within-organ gene co-regulation in the three primary cohorts; Figure S6: Per-patient detail for the six clinical/donor covariate comparisons; Figure S7: Marker evidence for the lung single-cell cell-type labels and composition by timepoint; Figure S8: Cell-type attribution in liver (single donor); Table S1: Antioxidant/reactive oxygen species gene panel with source database records (ros_panel.tsv); Table S2: Per-gene paired results for all eight cohorts (per_gene_paired_results.tsv); Table S3: Module-level results for all eight cohorts (module_all_cohorts.tsv); Table S4: Random-effects meta-analysis of the NFE2L2 axis and metallothionein modules across all eight cohorts (meta_analysis.tsv); Table S5: Leave-one-cohort-out sensitivity of the primary cross-organ comparison (leave_one_cohort_out.tsv); Table S6: Clinical and donor covariate stratification of the within-person module response (clinical_stratification.tsv); Table S7: Per-cell-type module response in the lung single-cell dataset (lung_sc_celltype_module.tsv); Table S8: Per-cell-type module response in the liver single-cell dataset (liver_sc_celltype_module.tsv); Table S9: Sensitivity of the random-effects pooled estimate to the two smallest contributing cohorts, with a distribution-free alternative (TableS9_pooling_sensitivity.tsv); Table S10: Independent recomputation of the headline numbers by a second, separately written script (TableS10_independent_verification.tsv); Table S11: Partition of the between-cohort heterogeneity into between-organ and within-organ components, with the pooled estimate for each organ (TableS11_organ_heterogeneity.tsv); Table S12: Stratification of the within-person module response by donor type in the three cohorts that record it (TableS12_procedural_strata.tsv); Table S13: Within-person change in *NFE2L2* and *KEAP1* transcripts against NFE2L2 target transcripts in the three primary cohorts (TableS13_nrf2_transcript_vs_targets.tsv); Table S14: Smallest standardised difference detectable with 80% power for each single-cell cell type (TableS14_sc_power.tsv); Table S15: Per-recipient reproducibility of the lung single-cell response (TableS15_sc_reproducibility.tsv).
