# Supplementary material for: Organ- and Cell-Specific Induction of the NFE2L2 Antioxidant Programme After Human Graft Reperfusion
Source: Biomolecules. 2026 Oct 13;16(10):1485. doi: 10.3390/biom16101485 (PMC13702131; doi:10.3390/biom16101485)
Supplement: Supplementary file 1 [file biomolecules-16-01485-s001.zip › biomolecules-4595014-supplementary.pdf]

biomolecules

MDPI

Supplementary Materials

Organ- and Cell-Specific Induction of the NFE2L2 Antioxidant Programme After Human Graft Reperfusion

Hyeonah Oh 1 and So Hyun Ahn 2,3,*

Supplementary Figures

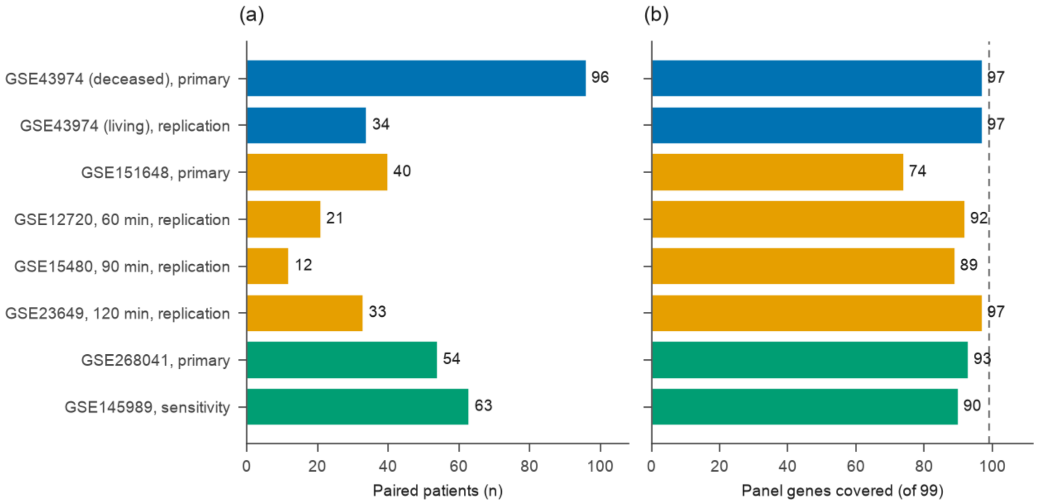

Figure S1. Cohort overview. (a) Paired patients contributed by each of the eight cohorts. (b) Antioxidant/ROS panel genes covered on each cohort's platform, out of the 99-gene panel; the dashed line marks full coverage.

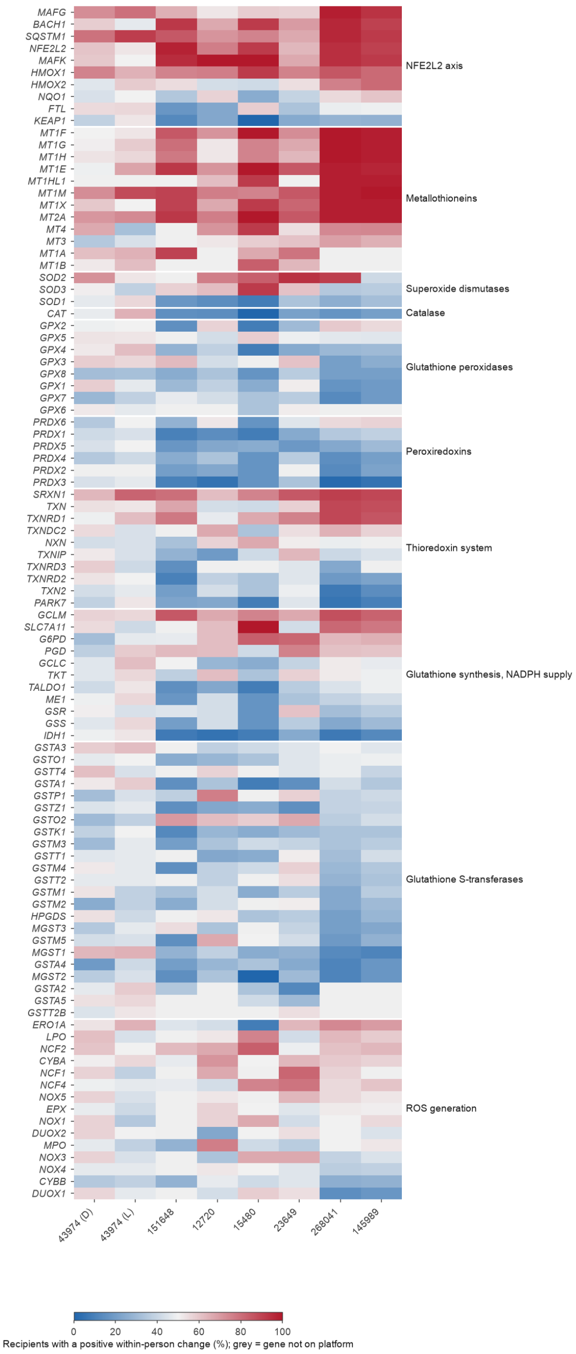

Figure S2. Within-person change for all 99 panel genes, in all eight cohorts. Colour encodes the percentage of recipients whose post-reperfusion value exceeded their own pre-reperfusion value, as in Fig. 1, now extended from the 71 genes shared by the three primary cohorts to the full panel and from the three primary cohorts to all eight; grey cells mark a gene not present on that cohort's platform.

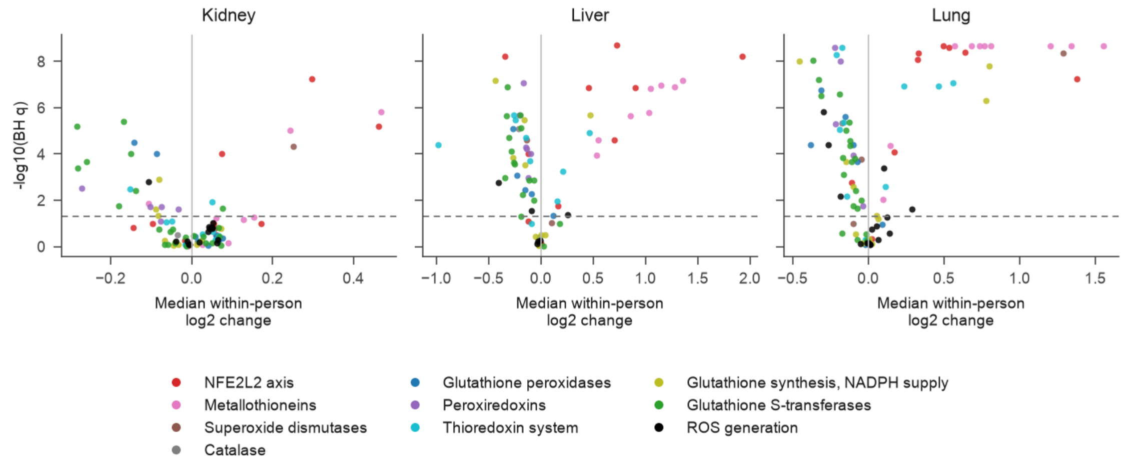

Figure S3. Volcano plots of the within-person response in each primary cohort (kidney, liver, lung). Each point is one panel gene, coloured by functional module; the dashed line marks a Benjamini-Hochberg false discovery rate of 0.05.

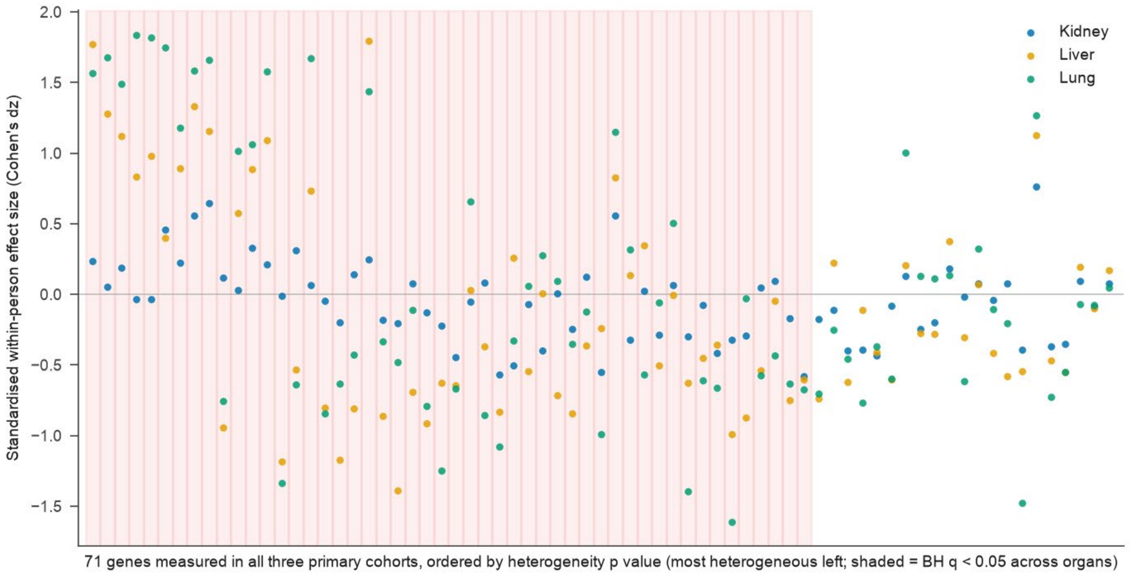

Figure S4. Cross-organ heterogeneity for the 71 genes measured in all three primary cohorts. Standardised within-person effect size (Cohen's d_z) is plotted for each gene in each organ; genes are ordered by the Kruskal-Wallis heterogeneity p value (most heterogeneous at left), and shading marks genes heterogeneous across organs at a Benjamini-Hochberg false discovery rate below 0.05.

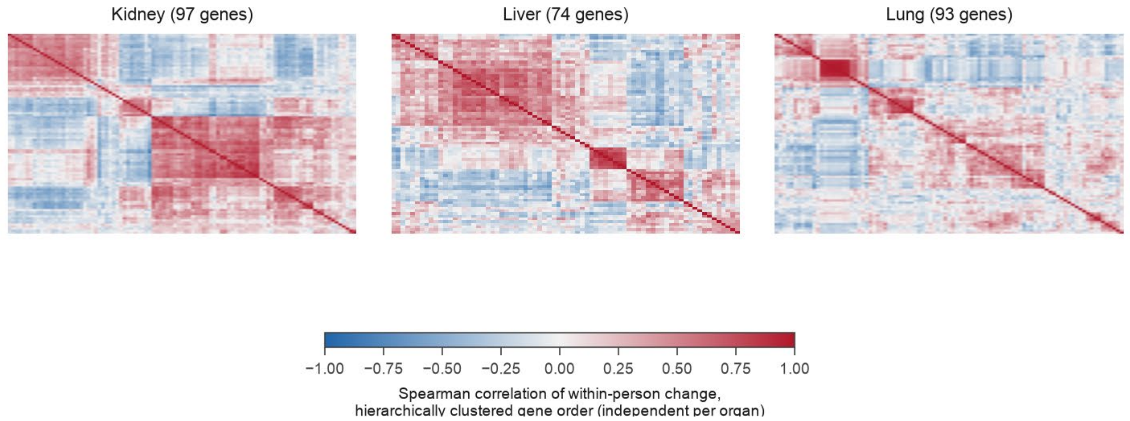

Figure S5. Within-organ gene co-regulation in the three primary cohorts. Spearman correlation of the within-person change between every pair of panel genes measured in that cohort, with genes ordered independently in each organ by average-linkage hierarchical clustering on one minus that correlation.

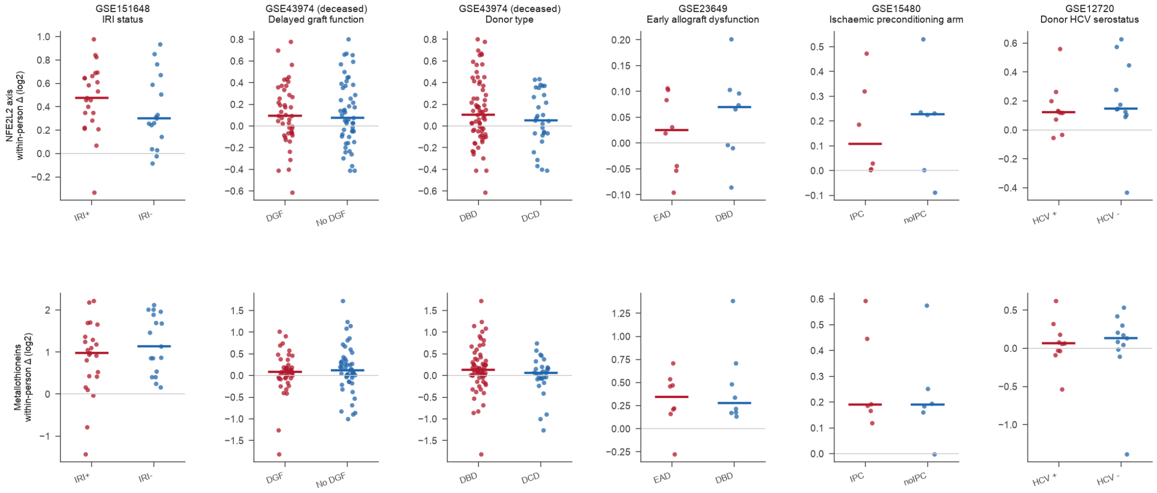

Figure S6. Per-patient detail underlying Fig. 7. Within-person module change ($\log_2$) for the NFE2L2 axis (top row) and the metallothioneins (bottom row), for every patient in each arm of each of the six covariates; horizontal bars mark the group median.

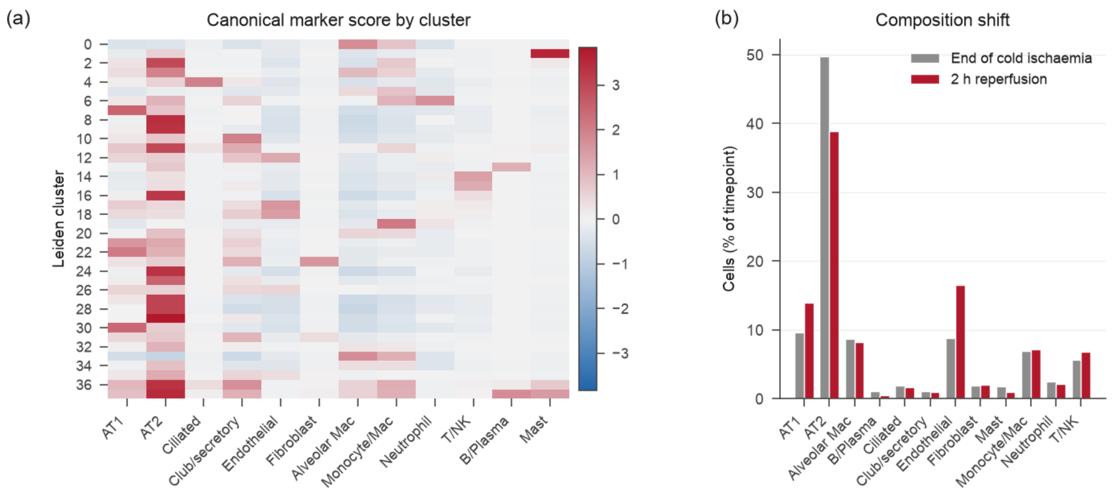

Figure S7. Evidence for the lung cell-type labels used in Fig. 8. (a) Mean canonical marker-set score for each Leiden cluster (every second cluster labelled); each cluster was assigned the best-scoring lineage, and clusters not reaching a fixed threshold were left unassigned. (b) Cell-type composition at each timepoint, as a percentage of the cells recovered at that timepoint. Composition differences between timepoints reflect both biology and differential recovery during tissue dissociation and are not interpreted as changes in absolute cell number.

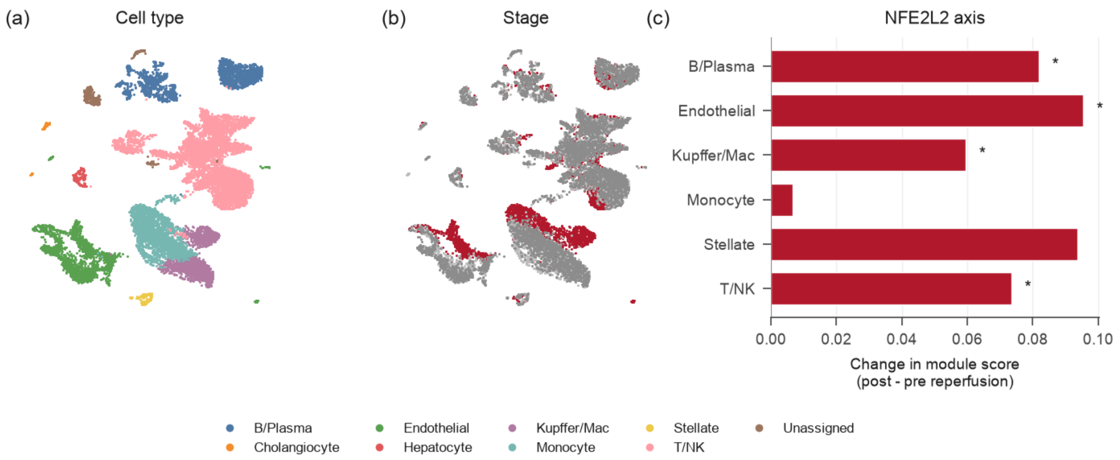

Figure S8. Cell-type attribution in liver, from a single donor sampled before procurement, at the end of preservation and 2 h after portal reperfusion (GSE171539; 14,228 cells after quality control). (a) UMAP coloured by cell type, (b) by stage, and (c) change in the NFE2L2 axis module score between post- and pre-reperfusion cells of the same cell type, with asterisks marking a Benjamini-Hochberg false discovery rate below 0.05. Cell types with fewer than 20 cells at either timepoint are not tested. Because this is one donor, the panel is shown as supporting cell-type attribution only and carries no patient-level replication.

Supplementary Tables

The following tables are supplied as separate tab-delimited files, named as given below, because each is too large to typeset in this document.

Table S1. Antioxidant/reactive oxygen species gene panel with the source database records justifying each gene. File: ros_panel.tsv (99 data rows).

Table S2. Per-gene paired results for all eight bulk cohorts. File: per_gene_paired_results.tsv (729 data rows).

Table S3. Module-level results for all eight bulk cohorts. File: module_all_cohorts.tsv (72 data rows).

Table S4. Random-effects meta-analysis of the NFE2L2 axis and metallothionein modules across all eight cohorts. File: meta_analysis.tsv (18 data rows).

Table S5. Leave-one-cohort-out sensitivity of the primary cross-organ comparison. File: leave_one_cohort_out.tsv (10 data rows).

Table S6. Clinical and donor covariate stratification of the within-person module response. File: clinical_stratification.tsv (12 data rows).

Table S7. Per-cell-type module response in the lung single-cell dataset (GSE220797). File: lung_sc_celltype_module.tsv (108 data rows).

Table S8. Per-cell-type module response in the liver single-cell dataset (GSE171539). File: liver_sc_celltype_module.tsv (54 data rows).
